# Genetic Analysis of Floral Symmetry Transition in African Violet Suggests the Involvement of Trans-acting Factor for *CYCLOIDEA* Expression Shifts

**DOI:** 10.3389/fpls.2018.01008

**Published:** 2018-08-15

**Authors:** Hui-Ju Hsu, Cheng-Wen He, Wen-Hsi Kuo, Kuan-Ting Hsin, Jing-Yi Lu, Zhao-Jun Pan, Chun-Neng Wang

**Affiliations:** ^1^Institute of Ecology and Evolutionary Biology, National Taiwan University, Taipei, Taiwan; ^2^Department of Life Science, National Taiwan University, Taipei, Taiwan

**Keywords:** *CYCLOIDEA*, *Saintpaulia ionantha*, zygomorphy, actinomorphy, *RADIALIS*, genotype–phenotype association, gene duplication, bilateral symmetry

## Abstract

With the growing demand for its ornamental uses, the African violet (*Saintpaulia ionantha*) has been popular owing to its variations in color, shape and its rapid responses to artificial selection. Wild type African violet (WT) is characterized by flowers with bilateral symmetry yet reversals showing radially symmetrical flowers such as dorsalized actinomorphic (DA) and ventralized actinomorphic (VA) peloria are common. Genetic crosses among WT, DA, and VA revealed that these floral symmetry transitions are likely to be controlled by three alleles at a single locus in which the levels of dominance are in a hierarchical fashion. To investigate whether the floral symmetry gene was responsible for these reversals, orthologs of *CYCLOIDEA* (*CYC*) were isolated and their expressions correlated to floral symmetry transitions. Quantitative RT-PCR and *in situ* results indicated that dorsal-specific *SiCYC1s* expression in WT *S. ionantha* (*SCYC1A* and *SiCYC1B*) shifted in DA with a heterotopically extended expression to all petals, but in VA, *SiCYC1s'* dorsally specific expressions were greatly reduced. Selection signature analysis revealed that the major high-expressed copy of *SCYC1A* had been constrained under purifying selection, whereas the low-expressed helper *SiCYC1B* appeared to be relaxed under purifying selection after the duplication into *SCYC1A* and *SiCYC1B*. Heterologous expression of *SCYC1A* in *Arabdiopsis* showed petal growth retardation which was attributed to limited cell proliferation. While expression shifts of *SCYC1A* and *SiCYC1B* correlate perfectly to the resulting symmetry phenotype transitions in F1s of WT and DA, there is no certain allelic combination of inherited *SiCYC1s* associated with specific symmetry phenotypes. This floral transition indicates that although the expression shifts of *SCYC1A/1B* are responsible for the two contrasting actinomorphic reversals in African violet, they are likely to be controlled by upstream trans-acting factors or epigenetic regulations.

## Introduction

**Figure 1:**
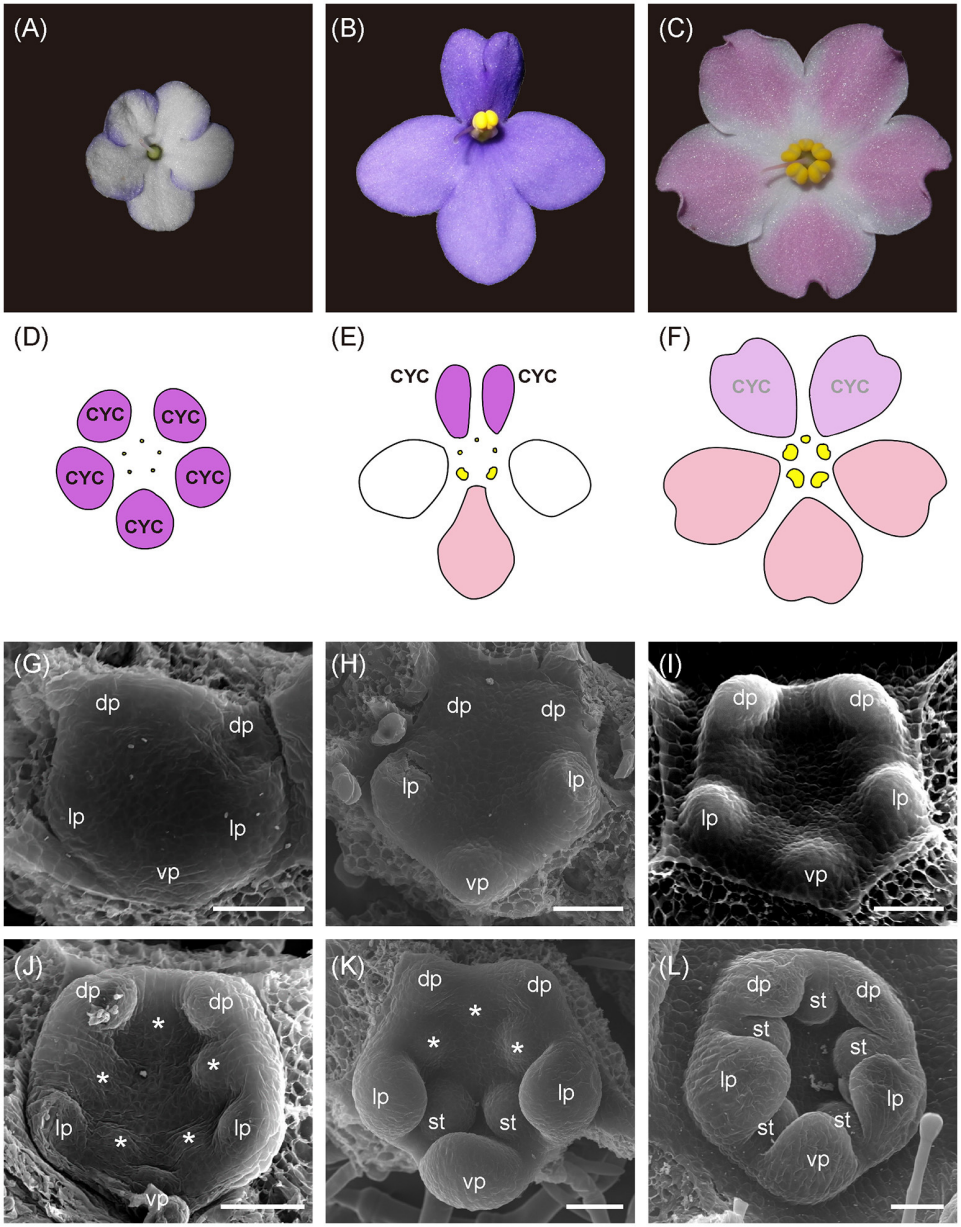
The photos, floral diagram, and SEM photos of flowers between zygomorphic wild type (WT) and its two actinomorphic mutants, dorsalized (DA) and ventralized peloria (VA). **(A,D)** The flower of DA is actinomorphic in that all five petals are small, similar to the size of dorsal petals (color in purple) in WT. No mature stamens can be seen; **(B,E)** In WT, two dorsal petals (purple) are smaller than the lateral (white) and ventral ones (pink). Two stamens are located in the ventral side between lateral and ventral petals; **(C,F)** The flower of VA is also actinomorphic in that all five petals are of larger size, similar to lateral and ventral petals of WT. Five mature stamens were developed in VA although the dorsal one is smaller. The SEM images of DA, WT, and VA flowers during petal initiation (**G–I**, stage 5) and stamen initiation (**J–L**, stage 6) stages. dp, dorsal petal; lp, lateral petal; vp, ventral petal; st, stamen; *, staminode (aborted stamen). Bars = 50 μm.

Variation in floral symmetry in horticultural species provides a great opportunity to study the molecular genetics of floral symmetry transition. Peloric mutations, the acquisition of actinomorphy (radial symmetry) by zygomorphic (with dorsiventral asymmetry) plants, have provided precious opportunities to examine the developmental genetics of floral symmetry transition. The African violet, *Saintpaulia ionantha* (Gesneriaceae), is ancestrally zygomorphic with a long cultivation history and is also famous for a great variety of reversion to actinomorphy cultivars. The zygomorphic wild type (WT) of *S ionantha* ssp. *velutina* (B.L. Burtt) I. Darbysh has flowers with two dorsal (adaxial) petals (dp) smaller than the three petals in lateral (lp) and ventral (abaxial) (vp) positions, (Figures 1B,K). It has only two stamens (positioned abaxially), while the adaxial and lateral stamens have been reduced to staminodes. One peloric (actinomorphic) cultivar *S. ionantha* ssp. *velutina* “little rick” (Figure 1C) of African violet arose in cultivation during the early 1950s as a single gene recessive (Reed, 1961). This peloric form differs from WT in having all five petals identical in shape and size, and all five stamens are fully functional (although the dorsal and lateral stamens are marginally smaller than the ventral ones) (Figure 1F). All five petals in this peloric form are mostly similar to the ventral petals of the wild type, and thus the peloric flowers appear to have lost their dorsal identity or acquired ventral identity (abbreviated as VA, ventralized actinomorphy, hereafter). Recently in flower markets, another peloric cultivar *S. ionantha* ssp. *gortei* (Engl.) I. Darbysh.“no stamen” has begun to grow in popularity owing to its numerous small-sized flowers and fused petals which form a somewhat unusually tubular corolla in a fully upright actinomorphy (Figure 1A). Interestingly, this peloria has all stamens aborted in mature flowers but it exists in cultivation as African violets can be easily propagated through leaf cuttings. This peloric cultivar differs from WT and VA in having all five petals similar to the small-sized dorsal petals of WT. “no stamen” is therefore a dramatically different peloria of African violet with dorsalized actinomorphy (abbreviated as DA, hereafter). With these cultivars, we can explore the flexibility of genetic control on floral symmetry transitions exerted by artificial selection.

The rise of two different peloric phenotypes, DA and VA, from the zygomorphic WT implies that the genes controlling dorsal and ventral identity may have been involved. In *Antirrhinum majus* (Snapdragon) and other zygomorphic flowering plants, *CYCLOIDEA* (*CYC*) gene is necessary to establish the dorsiventral flower axis (Luo et al., 1996, 1999). This *CYC* has been known to regulate cell proliferation (cell division) and cell expansion (Costa et al., 2005). In *Antirrhinum, CYC* is restricted to express in the dorsal part of the flower and is therefore responsible for the growth promotion of dorsal petals and growth retardation of the dorsal staminode (Luo et al., 1996; Clark and Coen, 2002). DICHOTOMA (*DICH*) is also expressed in the dorsal part of the flower, but its expression is largely confined to the inner half of each dorsal petal (Luo et al., 1999). Mutant phenotypes of *DICH*, therefore, only have slightly altered dorsal petal shape. This indicates that *CYC* has a stronger effect on altering dorsal petal morphology than *DICH* does. Mutation of both *CYC/DICH* results in actinomorphy, in which all petals switch to ventral identity (Coen and Nugent, 1994; Luo et al., 1996).

Floral symmetry transition has independently evolved among major angiosperm lineages and has been demonstrated to correlate with the shifts of *CYC* expression (Rosin and Kramer, 2009; reviewed in Busch and Zachgo, 2009; Hileman, 2014a). Most common mechanisms for floral symmetry transitions could be summarized into the spatial and temporal shifts in *CYC* expression (Spencer and Kim, 2018). Similar to *Antirrhinum*, reversals to actinomorphy attributable to a loss of dorsal-specific or asymmetrical expression of *CYC* were reported in *Tradescantia* (Commelinaceae, monocot, Preston and Hileman, 2012), in three Oleaceae species (early diverging Lamiales, Zhong and Kellogg, 2015), in two pollinator shifting Malpighiaceae lineages (Zhang et al., 2013), in *Lotus japonicas* double mutant (Fabaceae, Feng et al., 2006), and in tubular mutants of sunflower (Asteraceae, Chapman et al., 2012). In these cases, they are ventralized as all petals acquired ventral identity attributable to the loss of dorsal-specific *CYC*. On the other hand, reversals into DA attributable to the extension of *CYC* expression into all petals to acquire dorsal identity has been reported in *Cadia* (Fabaceae, Citerne et al., 2006), in two parallel Malpighiaceae lineages (Zhang et al., 2013), in petal-indistinct wind pollinated *Plantago lanceolata* (Reardon et al., 2009; Plantaginaceae, Preston et al., 2011), and in radially symmetrical Viburnum (Dipsacles, Howarth et al., 2011). The existence of both DA and VA in many angiosperm lineages indicates that the regulation of *CYC* can be very diverse.

Among Gesneriaceae species, reversals to actinomorphy from dorsalization and ventralization have both been described. Ventralized actinomorphy (VA) attributable to mutated *CYC* or loss of expression of *CYC* in the late petal development stage has been reported in *Sinningia speciosa*, and *Bournea* (Zhou et al., 2008; Hsu et al., 2015, 2017). On the other hand, DA has been reported in *Tengia* and occasionally in *Petrocosmea* hybrids in which *CYC* expression has extended to all petals (Pang et al., 2010; Yang et al., 2015).

It is worth mentioning that temporal (heterochronic) changes of *CYC* expression also trigger floral symmetry transition. The transient only dorsal expression of *TCP1* (*CYC*) in the early floral meristem stage, yet not persisting into later organogenesis stages, make it unable to trigger *Arabidopsis* and *Bournea* to develop zygomorphy (Cubas et al., 2001; Zhou et al., 2008). Another genetic mechanism involved in creating a peloric phenotype is epigenetic silencing caused by gene methylation. In the peloric form of *Linaria vulgaris* (toadflax), there were no *CYC* mRNA transcripts that could be detected in the flower (Cubas et al., 1999b), attributable to hyper-methylation of the gene. If this is the situation in African violet, we would expect an entire loss of *CYC* expression in flower buds.

Möller et al. (1999) first isolated partial *CYC* homologs from several Gesneriaceae species. In *Saintpaulia*, the *CYC* homologs exist as a pair of recently duplicated *CYC* paralogs (*SiCYC1A, SiCYC1B*) and both are direct orthologs of *Antirrhinum CYC* and *DICH* (Citerne et al., 2000; Wang et al., 2004a). Phylogenetic analysis indicated that *SiCYC1A* and *SiCYC1B* were recently duplicated in the lineage leading to the genus *Streptocarpus/Saintpaulia* complex. Whether or not *SiCYC1A* and *SiCYC1B* are involved in the establishment of zygomorphy and responsible for floral symmetry transitions in DA and VA peloria of African violet remains to be studied.

Paralogs of duplicated genes can evolve subfunctions or neofunctions through sequence and regulatory changes. In Gesneriaceae *CYC* (*GCYC*), duplication events were found to be exceptionally high (Citerne et al., 2000; Wang et al., 2004a; Zhong and Kellogg, 2015). Certain *GCYC* duplications were found that can either predate or postdate the divergence of the family, tribe, and genus level (Smith et al., 2004, 2006; Wang et al., 2004a). After gene duplication, different selective pressure may be exerted on each duplicate that thus generate functional specializations (Gao et al., 2008). For example, relaxed purifying selection (estimated by dN/dS, the ratio of non-synonymous to synonymous substitutions) was detected in TCP domains of *DICH* along the lineage along the snapdragon, but *CYC* was detected, persisting on purifying selection to maintain the function (Hileman and Baum, 2003). Other studies, however, reported that while one copy of *CYC* might have been positively selected after duplications, the other copy remained under purifying selection (Chapman et al., 2008; Zhong and Kellogg, 2015). Therefore, gene duplication of *CYC* is prevailing in many angiosperm lineages and perhaps diversifying selection on each copy may lead to their functional divergence (Bello et al., 2017). Indeed, duplicated *CYC* paralogs have been found differentiating their expression levels and patterns both spatially and temporally (Chapman et al., 2008; Jabbour et al., 2014; Zhong and Kellogg, 2015). Moreover, differential expressions of *CYC* after duplications have been found to be associated with multiple independent reversals to actinomorphy in Malpighiaceae species (Zhang et al., 2013). It would therefore be essential to know whether the *SiCYC* duplications in African violet have exerted heterogenous selection on each paralog and created expressional differentiation contributing to the transitions in floral symmetry.

Previous studies therefore suggest that the peloric forms of African violet could have arisen either by (1) a loss of function of *CYC*: gene mutations in coding sequence or the regulatory region thus the dorsally specific expression pattern of *SiCYC1s* (*SiCYC1A* and *SiCYC1B*) in the flower is lost, or (2) a gain of function of *CYC*: a shift in spatial or temporal expression thus *SiCYC1s* are symmetrically expressed along the flower's dorsiventral axis. To investigate the cause, we compared expression patterns of *SiCYC1s* in petals of wild type and both peloric forms (DA and VA) via quantitative real-time RT-PCR (qRT-PCR). We further use RNA *in-situ* hybridization (ISH) to detect any spatial shift of *SiCYC1s* mRNA transcripts within the early stages of flower buds.

The aim of this study is therefore to compare flower development between WT and two pelorias DA and VA, to characterize the expression differences of *SiCYC1s* in flowers of these cultivars, to detect signature of *SiCYC1s* selection following gene duplications, to investigate *SiCYC1s* functions by ectopically expressing them in *Arabidopsis*, and to validate the association between *SiCYC1s* genotypes and their corresponding floral symmetry phenotypes among F1s from genetic crossings between WT, DA, and VA. Together we shall realize whether various genetic mechanisms are involved in these two contrasting actinomorphic reversals within African violet.

## Materials and methods

### Plant materials

African violet WT, DA, and VA cultivars were propagated by leaf cuttings to maintain the same genetic unity. The original plants of WT and DA were provided by Dr. Cecilia Koo Botanic Conservation Center, Pingtung, Taiwan. At ChienKuo Holiday Flower Market, Taipei, Taiwan, VA were originally purchased. All materials were cultivated at continuously 24°C under 16 h light and 8 h darkness. Ploidy levels of all cultivars were checked by flow cytometry and the results showed that G1 peaks of all three cultivars are in relatively the same position. Hence all cultivars, WT, DA, and VA, perhaps share the same ploidy level (Figure S1).

### Flower development and petal cell measurement

Inflorescences of WT, DA, and VA were partially dissected to separate flower buds into stages of varying sizes (stage 1–7, Table S1, Figure S2). Buds were fixed in FAA (3.7% formaldehyde, 5% acetic acid, 50% ethanol in water) overnight and dehydrated through an ethanol/acetone series into 100% acetone dried. Material was then dried in a Hitachi HCP-2 critical point dryer. Dried buds were mounted with carbon conductive tape on 1.25 cm aluminum stubs, and further dissected. Stubs were sputter coated with gold-palladium using a Hitachi E101. Specimens were viewed using a scanning electron microscope (SEM, FEI Inspect S), and operating at 15 kV.

To compare the morphology of petal epidermal cell between WT, DA, and VA and transgenic *Arabidopsis* plants, petals of fully open flowers (stage 16, Table S1, Figure S3) were examined. Fresh tissues were mounted with carbon conductive tape on 1.25 cm aluminum stubs, then the stubs were dipped in liquid nitrogen and viewed using a Cryogenic scanning electron microscopy (Cryo-SEM) that was operating at 5 kV.

### Isolation of *CYC*-like genes homologs and reconstructed phylogeny

Parts of the *Saintpaulia SiCYC 1A* and *1B* sequences were first amplified with DNA and cDNA using primer pairs FS (5′-ATG CTA GGT TTC GAC AAG CC-3′) and R (5′-ATG AAT TTG TGC TGA TCC AAA ATG-3′) designed from highly conserved TCP and R domain as in Möller et al. (1999). This primer pair has been demonstrated to efficiently amplify all *CYC* copies from all major Gesneriaceae lineages (Wang et al., 2004a). Partial sequences obtained were then cloned for the identity of *SiCYC1A* and *SiCYC1B*. To get full length sequence of these *SiCYC1s*, we used the genome walking technique to amplify the remaining 5′-end upstream flanking region and 3′ RACE for 3′-end sequences, as previously described (Wang et al., 2004b).

For phylogeny reconstruction, the nucleotide sequences of available Genbank *GCYC* sequences (Table S2) and those from *Antirrhinum* were isolated and aligned with *SiCYC1s* based on their amino acid sequences using MAFFT (https://mafft.cbrc.jp/alignment/server/) and manually adjusted. The maximum likelihood (ML) analysis was conducted in RAxML version 8 (Stamatakis, 2014). Twenty heuristic searches under the General Time Reversible (GTR) + γ nucleotide substitution model with no partition were performed and the congruence was checked manually. One thousand bootstrap trees were calculated and consensus values >70% were mapped to the best tree. The Bayesian inference (BI) tree was reconstructed by BEAST2 (Bouckaert et al., 2014) under the same substitution model with 100,000,000 generations with the samplings every 5,000 generations. The stability of final posterior probability (ESS state) was confirmed in Tracer 1.6 software. If the BI tree topology was congruous with the ML tree, the posterior probabilities >0.80 were mapped to the backbone of ML tree at the corresponding nodes.

### RNA extraction and quantitative real-time RT-PCR

Total RNA of dorsal, lateral, and ventral petals of stage 9, 12, and 15 of flowers was separately extracted using Trizol (Invitrogen, Carlsbad, CA, USA). Additional phase separation step by acid phenol: chloroform: IAA (25: 24: 1, pH 4.5) was added before the precipitation step to aid the removal of DNA. The MMLV reverse transcriptase (Invitrogen) was used to synthesize cDNA from total RNA with a mixture of oligo dT primers (5′-TTT TTT TTT TTT TTT TTV-3′) and random hexamer. Quantitative real-time PCR (qPCR) was performed with the KAPA SYBR FAST qPCR kit (KAPA Biosystems, Woburn, MA, USA) in Bio-Rad CFX real-time PCR machine (Bio-Rad, Hercules, CA, USA). qPCR primers for each *SiCYC1s* copy are listed in Table S3. The melting curve and the obtained threshold cycle (Ct) values were analyzed in CFX Manager 3.0 (BioRad, 2013). All the reactions were performed in triplicate and had NTC (no template control) and NRC (no reverse transcription control) to exclude contamination from chemicals and DNA contamination from RNA samples, respectively. Housekeeping genes in African violet for the purpose of internal control to calibrate equal amounts of RNA as reference were *18S rRNA* (for WT, DA, and VA comparison) or β*-actin* (actin 7) (for comparison in flowers of F1 hybrids between WT × DA). The expression amount was calculated by the formula: ${\text{E}}_{\text{ref}}^{\text{ct}}$/E ${\text{E}}_{\text{exp}}^{\text{ct}}$ (E = 1 + efficiency value). The efficiency value of each primer pair was calculated from the slope of the standard curve using the formula: efficiency value = 10^−1/slope^. The reason why the internal control was switched to β*-actin* in F1 hybrids is due to the *SiCYCs* expression levels being low, the 18S expression levels being too high, and thus causing difficulty in the quantification of *SiCYCs'* relative expression amount.

### *In situ* hybridization for *SiCYC1s*

mRNA ISH was carried out by the following methods by Wang et al. (2008). To synthesize gene-specific RNA probes, the partial sequence of *SiCYC1A* (252 bps in R domain) was cloned into the pGEM-T easy vector system (Promega, Fitchburg, WI, USA), using primers pSiCYC1A_DFA_F1 (5′-GAT CCA GCA CAG AGT GCA TCA AC-3′) andpSiCYC1A_HQS_R1 (5′-CGA CTG GTG GTG CCT CAG-3′). These plasmids were then digested with SpeI (Thermo Fisher Scientific Inc., Waltham, MA, USA). Digoxigenin (DIG)-labeled antisense RNA probes and sense RNA probes were synthesized with DIG-dNTP (Roche Diagnostics GmbH, Basel, Schwelz) by T7 RNA polymerase and SP6 RNA polymerase, separately, using digested plasmid as the template. The plant material was fixed in 4% paraformaldehyde and processed for paraffin embedding and sectioning. The sections were hybridized with RNA probes and hybridization signals were detected by Anti-DIG-AP (Roche Diagnostics GmbH) with NBT/BCIP as the substrate. Hybridization was visible as purple to blue signals. The sections were observed under a BX51 (Olympus) bright field microscope. Sense RNA probes were used for negative control.

### Detection of selective pressure differences between *SiCYC1A* and *SiCYC1B*

**Figure 2:**
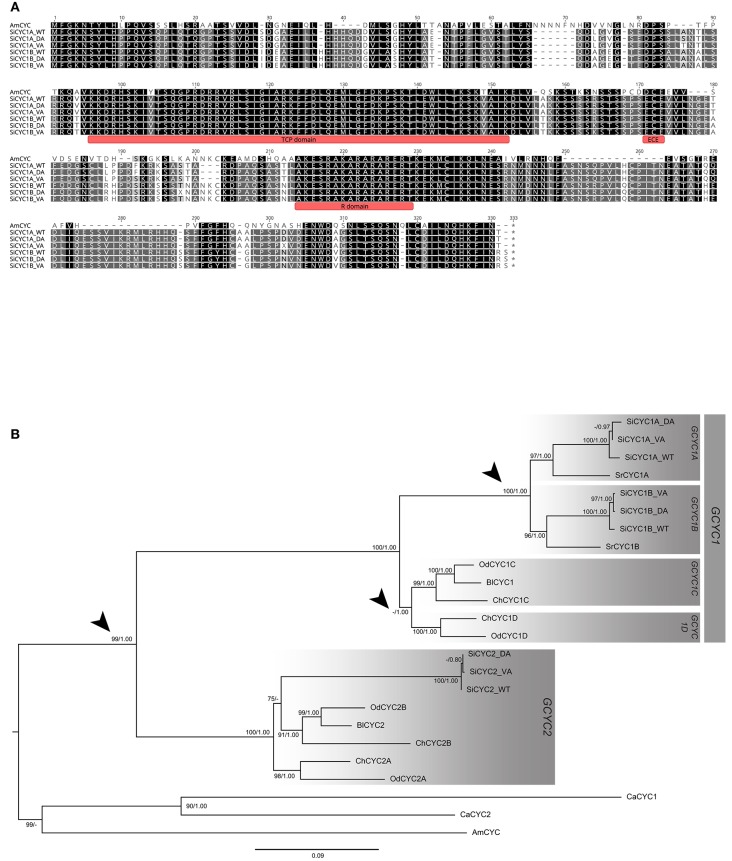
Alignment and the phylogeny of African violet SiCYC1A and *SiCYC1B* with selected Gesneriaceae GCYC homologs. **(A)** The alignment of *SiCYC1A* and *SiCYC1B* with *AmCYC* (snapdragon), with TCP, ECE, and R domains is indicated. **(B)** The ML tree with *AmCYC* as outgroup. The topology indicates that the evolution of Gesneriaceae *GCYC* homologs contains multiple duplication events (arrow heads). In particular, the duplication before the divergence of genus *Streptocarpus* (including African violet) which gave rise to *GCYC1A* (*SiCYC1A*) and *GCYC1B* (*SiCYC1B*) becomes the major paralogs involving floral symmetry transitions in African violet. ML bootstrap supports (> 70%) and BI posterior probabilities (> 0.80) are labeled.

To elucidate whether different selective pressures has affected the evolution of *SiCYC1A* and *SiCYC1B* after their duplication, molecular evolution of *GCYC* duplicates in Gesneriaceae was investigated with codeml from the PAML package v.4.4 (Yang, 2007). From previous *GCYC* phylogeny (Wang et al., 2004a) and our study (Figure 2), *SiCYC1A* and *SiCYC1B* each is grouped with other *GCYC1A* and *GCYC1B* genes, respectively, from *Streptocarpus*/*Saintpaulia* species. Therefore, *SiCYC1A* and *SiCYC1B* from African violet were aligned with available *Streptocarpus GCYC1A* and *GCYC1B* from NCBI (accession numbers in Table S4). *GCYC1C* genes from Gesneriaceae species were selected as the outgroup on account of their closest relationship with *GCYC1A* and *GCYC1B* (Wang et al., 2004a).

To find evidence of divergent or positive selection along *GCYC1A* (*SiCYC1A*) and *GCYC1B* (*SiCYC1B*) lineages, models of different evolutionary scenarios of selective pressures were analyzed. The tree topology was thereafter unrooted and applied to the evaluation of selective pressure at molecular level by ω value (K_N_/K_S_) using CODEML in PAML v4.0 (Yang, 2007). We especially focused on detecting whether selective pressure changes before and post duplication event occurred. To achieve this goal, we modified the duplication ML model from Bielawski and Yang (2003) to test the change in selective regime before and after gene duplication. All gaps in the sequence alignment were treated as missing data (marked as “?” in the alignment in CODEML) to retain the information from these sites. Branch models allowing ω values varying on different lineages were designed according to evolutionary scenarios. Thus, *GCYC1A* and *GCYC1B* were separately assigned as foreground in two-ratio branch models for testing shifts in selective pressures (ω values) along each duplicate (Table 1). All the two-ratio models were tested against the general one-ratio null model of constant selective pressures. A branch site three ratio model was further compared with *GCYC1A* and *GCYC1B* as different foregrounds in the same model to test the hypothesis of lineage-specific selection. The three-ratio model was tested against the “post-duplication” model. Site model was set up for testing the selective pressures acting on certain portions of sites. Branch-site models were also implemented to increase the detectability in previous scenarios in branch models with the consideration of site proportion. Two Clade Model C models with the foreground as *GCYC1A* and post-duplication lineages were tested against the null model m2a_rel (Weadick and Chang, 2011). The further “lineage-specific” model was compared to the “post-duplication” model to determine whether the two duplicate lineages have difference in selective pressure (Table 1).

**Table 1:** Parameter estimates and likelihood scores of the nested branch and branch-sites models for detecting selective constrains between *GCYC1A* and *GCYC1B*.

**Clade**	**Model**	**n.p**.	**Log Likelihood (lnL)**	**2ΔL^a^**	***P*-value^b^**	**Estimates of ω value**
**BRANCH MODEL**						
Null	One-ratio	39	−2898.3994			ω_0_ = 0.3690
*GCYC1A* as foreground	Two-ratio	40	−2895.4538	5.8911	0.015^*^	ω_0_ = 0.4168, ω*_*GCYC*1*A*_* = 0.2083
*GCYC1B* as foreground	Two-ratio	40	−2898.3696	0.05958	0.8	ω_0_ = 0.3640, ω*_*GCYC*1*B*_* = 0.3884
*GCYC1A* + *GCYC1B* as foreground (post-duplication)	Two-ratio	40	−2896.9931	2.8125	0.094	ω_0_ = 0.4262, ω_post−duplication_ = 0.2943
*GCYC1A*, *GCYC1B* as foreground (lineage-specific)	Three-ratio	41	−2895.4048	3.1769	0.075	ω_0_ = 0.4260, ω*_*GCYC*1*A*_* = 0.2085, ω*_*GCYC*1*B*_* = 0.3909
**BRANCH-SITE MODEL (CLADE MODEL C)**						
M2a_rel (null)	One-ratio	42	−2883.5782			*P1* = 0.3177, ω_P1_ = 0.0182; *P2* = 0.1128, ω_P2_ = 1.0000; *P3* = 0.4789, ω_P3_ = 0.4789
*GCYC1A* as foreground	Two-ratio	43	−2879.2689	8.6185	0.0033^***^	*P1* = 0.5190, ω_0_ = 0.1030, ω*_*GCYC*1*A*_* = 0.1030; *P2* = 0.0473, ω_0_ = 1.0000, ω*_*GCYC*1*A*_* = 1.0000; *P3* = 0.4338, ω_0_ = 0.8369, ω*_*GCYC*1*A*_* = 0.2075
*GCYC1A* + *GCYC1B* as foreground (post-duplication)	Two-ratio	43	−2880.7007	5.755	0.016^*^	*P1* = 0.5148, ω_0_ = 0.1108, ω_post−duplication_ = 0.1108; *P2* = 0.2042, ω_0_ = 1.0000, ω_post−duplication_ = 1.0000; *P3* = 0.2811, ω_0_ = 0.9178, ω_post−duplication_ = 0.1472
*GCYC1A*, *GCYC1B* as foreground (lineage-specific)	Three-ratio	44	−2879.1924	8.7715	0.012^*^	*P1* = 0.5081, ω_0_ = 0.0990, ω*_*GCYC*1*A*_* = 0.0990, ω*_*GCYC*1*B*_* = 0.0990; *P2* = 0.5081, ω_0_ = 1.0000, ω*_*GCYC*1*A*_* = 1.0000, ω*_*GCYC*1*B*_* = 1.0000; *P3* = 0.4241, ω_0_ = 0.8520, ω*_*GCYC*1*A*_* = 0.1784, ω*_*GCYC*1*B*_* = 0.7077

a *Models were compared by the double value of the difference in likelihood (2ΔL). Each likelihood ratio was calculated in comparison of alternative models and the suitable null model described in the main text*.

b *The statistical significance of models were tested by Likelihood Ratio Test (LRT). P-values were calculated from the 2ΔL of each comparison in the Chi-Square distribution with the degree of freedom equals to difference of parameters (n.p.) between compared models. Significant p-value is highlighted in bold*.

* *< 0.05*;

**< 0.01;

*** *< 0.001*.

### Genotyping *SiCYC* alleles by PCR-RFLP

To find out the association between *SiCYC1A* and *SiCYC1B* alleles and floral symmetry phenotypes among F1 individuals, PCR-based restriction fragment length polymorphism (RFLP) was conducted. This PCR-RFLP procedure was divided into 4 parts: (1) isolation of genomic DNA, (2) performing a standard PCR, (3) digestion with appropriate enzymes, and (4) resolution digest of PCR products. The enzymes used for digesting *SiCYC1A* and *SiCYC1B* PCR product were *Aat*II (#ER0991, Thermo) and *Pst*I(#ER0611, Thermo), respectively. The two chosen enzymes were determined by the allelic variation of DA *SiCYC1A* and *SiCYC1B*. The protocol for digestion of PCR products after amplification followed the manufacturer's instructions.

### Construction of *SiCYC1A* transgenic plants in *Arabidopsis*

The overexpression construct of *SiCYC1A*WT allele, p35S::*SiCYC1A*^*w*^:c-Myc (hereinafter referred to as *35S::SiCYC1A*^*W*^), and *SiCYC1A*VA allele, p35S::*SiCYC1A*^*V*^:c-Myc (hereinafter referred to as *35S::SiCYC1A*^*V*^) was engineered to pK2GW7.0 from the Gateway® system. Firstly, the sequences of *SiCYC1A* were amplified from cDNA by KAPA HiFi PCR Kits (KAPA biosystems, KK2501). The A-tailing PCR products were cloned into pGEM®-T Easy Vector (Promega, A1360). Using the extracted plasmid as the template, the secondary PCR reaction with c-Myc fusion primers was carried out by KAPA HiFi PCR Kits. The products were further purified for cloning into pCR®8/GW/TOPO® TA Cloning Kit. Owing to the fact that both pCR®8/GW/TOPO® Donor vector and pK2GW7.0 Destination vector contain the same resistant gene (spectinomycin), the Donor vector must be linearized by Pvu I to destroy the self-replication ability in *E. coli* cells. The gel purified products were then ready for LR reaction using LR Clonase^TM^ II Enzyme Mix (Invitrogen) and the products were subsequently transformed into *E. coli* for amplification. The inserts in *E. coli* plasmids were cut by a restriction enzyme for gel validation, the correct size of the vectors, and the desired insertion size. The interchangeable regions, flanked by attB1 and attB2 sites, were sequenced to avoid any mutation or frame shift. The sequence confirmed vectors were then transformed into *Agrobacterium tumefaciens* GV3101 by electroporation for subsequent *Arabidopsis* transformation.

The *Arabidopsis* floral dip transformation protocol was followed by Clough and Bent (1988). The floral dip procedure was repeated once within the 8 days for obtaining more transformed seeds. Putative transgenic *Arabidopsis thaliana* T1 seedlings were selected by antibiotic kanamycin and PCR confirmed with T-DNA insertion primers *SiCYC1A*-F/R (5′-ATG TTT GGC AAG AAC TCG TAC CTT C-3′/5′-TTA CGT ATT GAT GAA TTT GTGCTG ATC C-3′) and NPTII-F/R (5′-TCA GAA GAA CTC GT CAA GAA-3′/5′-AAC AAG ATG GAT TGC ACG CA-3′). The transformed seedlings (T1) were selfed into T2 plants to document the phenotypic effect of each construct. For each allele construct, 3–7 independent T2 transgenic individuals of overexpressing *SiCYC1A*^*W*^ and *SiCYC1A*^*V*^, together with T1 plants, were summarized for the phenotypic effects on petal morphology. The mRNA expression level of *SiCYC1A* in T1 and T2 transgenic plants was verified by RT-PCR.

## Results

### Developmental transitions to actinomorphy occurred early at petal and stamen initiation stages

To figure out the developmental differences of floral symmetry between *Saintpaulia* cultivars, the flower buds from early to late stages of WT, DA, and VA were examined by SEM. The stage definitions of flower development in African violet (Table S1) basically followed the work by Harrison et al. (1999). The SEM results showed that the floral symmetry transitions between actinomorphic cultivars and wild type were evident as early as petal and stamen primordia initiation stages. In WT flowers, dorsal petals and adaxial stamen primordia initiated late and smaller than lateral and ventral ones (stages 5–6, Figures 1H,K; Figures S2Q,T). Along the developmental stages, dorsal petals always retained smaller than lateral and ventral petal primordia (Figures S2W, S3B). This contributes to the development of flower zygomorphy with two smaller dorsal petals and three larger lateral and ventral petals (Figure 1B). The three adaxial-side staminodes also retained smaller than the two abaxial stamens (Figures S2T,Z) and finally aborted thus only two abaxial stamens matured (Figures 1B,E). The petal aestivation showed that two lateral petals enfold the dorsal and ventral petals (Figure S2W).

However, in DA, all petal and staminode primordia were initiated at the same time (Figures 1G,J; Figures S2P,S) and the whole bud remained in small size when compared to other cultivars along floral development (Figures S2V, S3A). Eventually all 5 staminodes were completely aborted (Figures 1A,D). On the other hand, in VA all petal and stamen primordia also initiated together at the same time (Figures 1I,L; Figures S2R,U) but they enlarged faster along floral development (Figures S2X, S3C). All 5 stamens were fully developed when mature although there is a residual zygomorphy where the adaxial stamen is slightly smaller in size than the two lateral ones whereas the ventral two are the largest (Figures 1C,F). The aestivation of both DA and VA cultivars was random (Figures S2V,X).

### Cell proliferation but not cell growth cause dorsiventral petal size difference in african violet

To clarify whether the dorsiventral petal size (area) difference was caused by differential cell proliferation or cell growth, epidermal cell size and morphology between dorsal, lateral, and ventral petals in WT, DA, and VA were compared at anthesis (stage 16) via SEM (Figure S4). The SEM photos showed that the size and morphology of petal cells between all petals and all cultivars (WT, DA, and VA) were not obviously different. This raises the possibility that the dorsiventral petal size difference was perhaps not owing to cell growth differences but cell proliferations.

We further measured the petal area (mm^2^) and cell size from SEM photos through ImageJ software. Both petal area and cell size were converted to a ratio between ventral petal and dorsal petal (V/D, Table S5). In WT, the petal area of ventral petals was 2.16 times larger than dorsal petals. But in DA and VA, the ratios of petal area between ventral and dorsal petals was close to 1 (1.07 and 1.11, respectively). When comparing cell size differences between ventral and dorsal petals, the ratios (V/D) are about the same (0.87–1.17) across all cultivars, no matter where the samples were selected from proximal or distal petal parts. Given the fact that cell size has no difference between ventral/dorsal petals of all cultivars, the larger dorsal petal area in WT must be the result of the difference of cell number (cell proliferation) rather than that of cell size (cell growth).

### *CYC* homologs identified and duplications revealed from phylogeny

To identify putative genes involved in floral symmetry in African violet, *CYC*-like genes (*SiCYC1A, SiCYC1B, SiCYC2*) were isolated in WT, DA, and VA (Figure 2A). Maximum likelihood (ML) and BI trees were reconstructed to identify the putative *CYC*-like homologs (Figure 2B). Sequences from actinomorphic peloric cultivars, DA and VA, were not found to have any frameshift mutation (all indels were in multiples of three nucleotides) or stop codons.

Three *CYC* homologous (*GCYC1A, GCYC1B*, and *GCYC2*) were isolated from WT, DA, and VA of African violet (Figure 2A). The full length ORFs are 954 bp (*SiCYC1A*) and 969 bp (*SiCYC1B*), and the length is conserved between the WT, DA, and VA peloria except some single-nucleotide polymorphism (SNP) sites exists between them (Figure 2A). The divergence between *SiCYC1A* and *SiCYC1B* are 12.8% at the amino acid level and 9.7% at the nucleotide level. *SiCYC1A* and *SiCYC1B* contain an apparent TCP domain (Figure 2A) and an additional *CYC/TB1* R domain (Cubas et al., 1999a). The 58 amino acid TCP domain contains a basic-Helix-Loop-Helix (bHLH) structure predicted from PSIPRED protein structure analysis. This indicates *SiCYC1A* and *SiCYC1B* are putative transcription factors utilizing bHLH structure in the TCP domain to facilitate DNA binding with downstream genes or involving dimerization. The cultivars of WT and VA are homozygous for *SiCYC1A* and *SiCYC1B*. The alleles of WT are denoted as *SiCYC1A*^*WT*^ and *SiCYC1B*^*WT*^, for VA, *SiCYC1A*^*VA*^, and *SiCYC1B*^*VA*^ (Figure 2A). However, DA cultivar is heterozygous for *SiCYC1A* and *SiCYC1B*, containing one allele specific to DA (*SiCYC1A*^*DA*^ and *SiCYC1B*^*DA*^) with another allele from WT (*SiCYC1A*^*WT*^ and *SiCYC1B*^*WT*^). The allelic divergence of *SiCYC1A* between WT, DA and VA is small, that is, 0.9% at the amino acid level (3 of 318 are different) with 12 nucleotide substitutions in 954 bp (including some synonymous substitutions). The allelic divergence of *SiCYC1B* between WT, DA, and VA are even minute, that is, 0.3% at the amino acid level (1 of 323 are different) with 7 nucleotide substitutions in 954 bp (synonymous substitutions included as well).

In the Bayesian phylogenetic tree with *Antirrihinum CYC* as the outgroup including available old world Gesneriaceae, *GCYC*s from GenBank, *SiCYC1A*, and *SiCYC1B* (together with *SrCYCs* from *Streptocarpus rexii*) were nested in their own clade with strong branch support (*BS* = 100%) (Figure 2B). In fact, when we added more available genus *Streptocarpus/Saintpaulia GCYC* sequences in, every species contains both *GCYC1A* and *GCYC1B* clade duplicates (see below). These indicate that *SiCYC1A* and *SiCYC1B* are duplicated paralogs (*GCYC1A* and *GCYC1B*) that predated the origin of *Streptocarpus/Saintpaulia* species.

### Expression shifts of *SiCYC1s* correlates to developmental reversals to actinomorphy in DA and VA peloria

**Figure 3:**
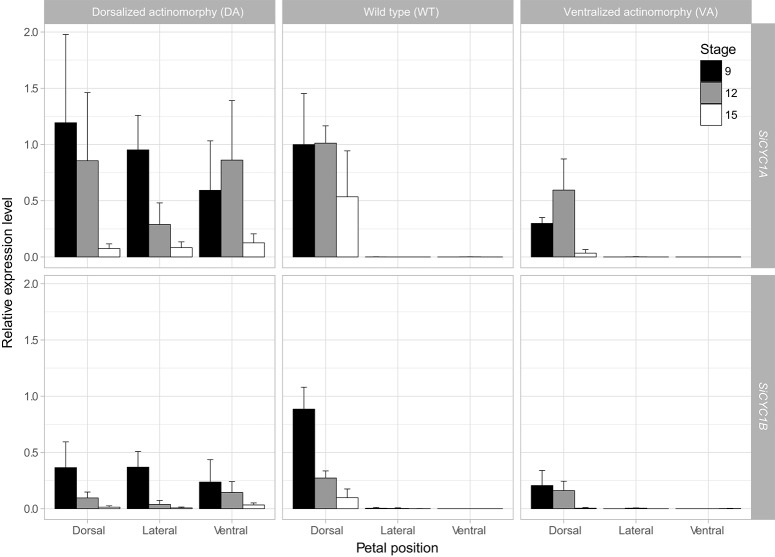
qRT-PCR expression of *SiCYC1A* and *SiCYC1B* in dissected petals of African violet. The expressions levels are compared between dorsal, lateral, and ventral petals at stage 9 (black bar), 12 (gray bar), and 15 (white bar) of DA, WT, and VA. *SiCYC1A* and *SiCYC1B* both express in all petals (dorsal, lateral, and ventral) of DA, but in WT their expressions are restricted to dorsal petals. *SiCYC1A* and *SiCYC1B* expressions are weak in VA but are still confined to dorsal petals. Each bar represents three biological repeats (mean ± SD). The relative expression levels are normalized to *18S rRNA*, and the fold change of expressions are calculated relative to *SiCYC1A* expression level of stage 9 dorsal petals of WT.

The expression patterns of these *SiCYCs* were compared between petal parts (i.e., dorsal, lateral, and ventral) among all cultivars in trying to associate the possible expression shifts to the transitions of floral symmetry. The quantitative (q)RT-PCR results revealed that the expression of *SiCYC1s, SiCYC1A*, and *SiCYC1B* were shifted corresponding to apparent floral symmetry reversals in DA and VA pelorias. In zygomorphic WT, expressions of *SiCYC1A* and *SiCYC1B* were both restricted to the dorsal petals (Figure 3, middle panel). But in DA peloria, expression of both *SiCYC1A* and *SiCYC1B* extended to all dorsal, lateral, and ventral petals (Figure 3, left panel). This *CYC* ubiquitous expression correlates with the development of 5 small-sized petals and growth retardation of all stamens in DA. On the other hand, in VA peloria, although *SiCYC1A* and *SiCYC1B* expressions are 50% lower than those in WT their expressions are still restricted to dorsal petals (Figure 3, right panel). The reduced expression of *SiCYC1s* in VA correlates with its 5 equal sized enlarged petals and fully developed stamens. Besides, the transcript levels of *SiCYC1A* (Figure 3, top row) were always higher than those of *SiCYC1B* (Figure 3, bottom row). The changes of expression patterns in both *SiCYC1A* and *SiCYC1B* among WT, DA, and VA were most evident at stage 9 (the petal whorl starts to enlarge as the size of sepal whorl, Figure S3). The transcripts of both *SiCYC1A* and *SiCYC1B* were declined during later flower development toward anthesis (stage 15) in all cultivars (Figure 3). The expression patterns of *SiCYC2* were not examined since the transcript of *SiCYC2* could not be detected in any part of the inflorescence along different stages.

### *SiCYC1s* expression shifts also observed in early bud stages of peloria by ISH

We further used RNA ISH to determine the exact *SiCYC1s* transcript locations within flower buds in early flower development (floral meristem, petal and stamen primordia initiation, and early differentiation stages) between WT, DA, and VA. Owing to the high similarity of nucleotide sequences between *SiCYC1A* and *SiCYC1B* (93.7% identity, 16 sites differences in 252 bps probe), we could not rule out that the anti-sense probe for *SiCYC1A* may also have hybridized to *SiCYC1B*. Nonetheless, *SiCYC1s* transcripts locations within the flower buds detected by ISH showed similar patterns as qRT-PCR results did.

**Figure 4:**
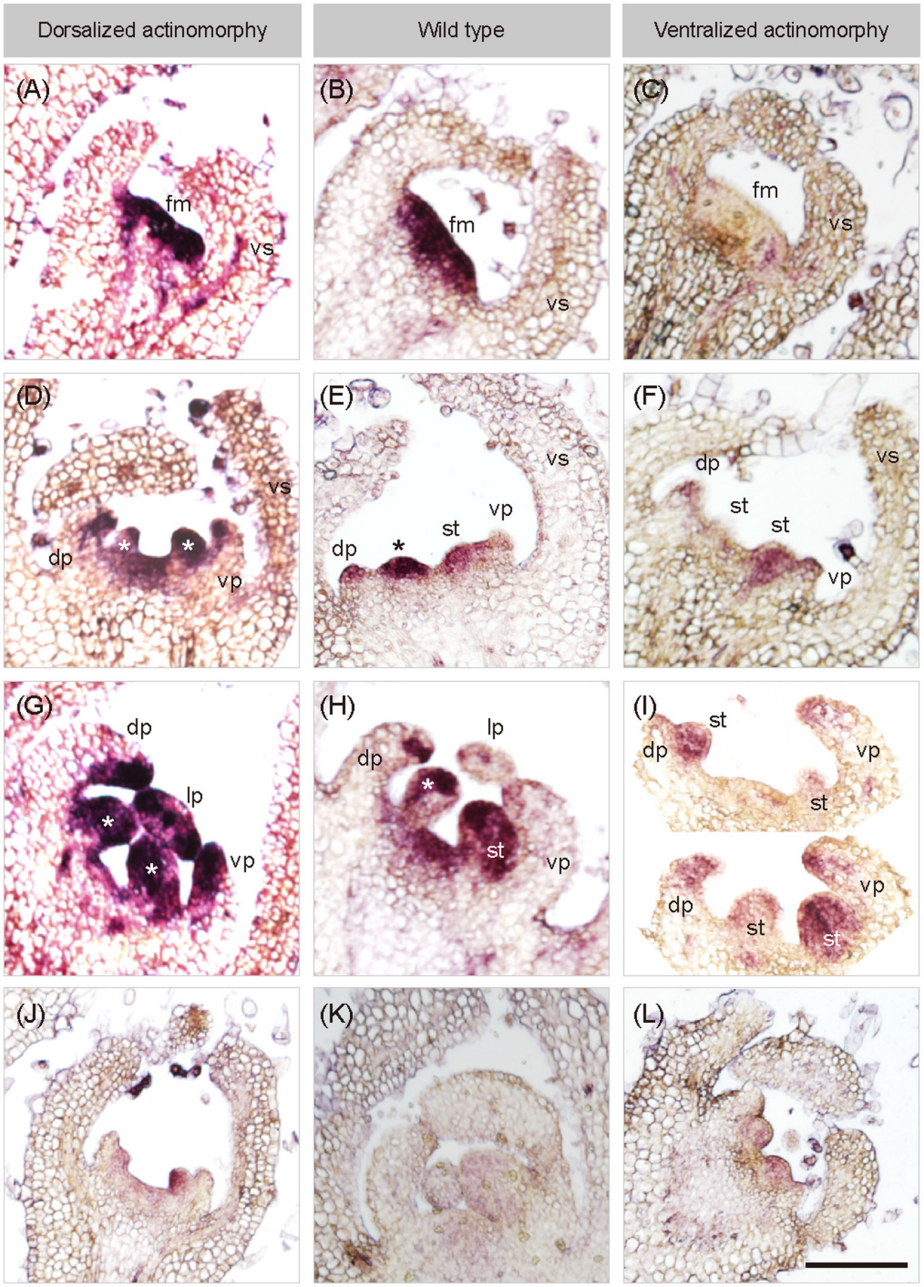
*In situ* hybridization of *SiCYC1A* in flower buds of African violet. Longitudinal sections on early flowering stages of all cultivars hybridized with antisense probes of *SiCYC1A:* stage 4 **(A–C)**, stage 5 **(D–F)** and stage 6 **(G–I)**. The purple color represents the *in-situ* signals of *SiCYC1A* mRNA. *SiCYC1A* has early expression in the entire floral meristem of DA and WT **(A,B)** but is absent in VA **(C)**. During floral organ initiation **(D–F)**, *SiCYC1A* persists in inner whorls of the flower including the dorsal (dp) and ventral petal primordia (vp) of DA **(D)**. **(E)** But in zygomorphic WT, *SiCYC1A* confines more to the dorsal petal primordia (dp) than the ventral one. **(F)** In VA, weak *SiCYC1A* signals start to acuminate at tips of both dorsal and ventral petal primordia. **(G)** Later in floral organ differentiation, *SiCYC1A* can be detected in all petals (dp, lp and vp) of DA. **(H)** But in WT, *SiCYC1A* is largely restricted to dorsal petals (dp). **(I)** In the petal whorl of VA, a weak but slightly stronger *SiCYC1A* is evident in dorsal petals (dp) when compared to ventral petals (vp). The sense probe controls were shown in **(J–L)**. The cross sections of *ISH* are shown in Figure S5. **fm**, floral meristem; **vs**, ventral sepal; **dp**, dorsal petal; **lp**, lateral petal; **vp**, ventral petal; **st**, stamen. *, staminode. Scale bar = 100 μm.

From ISH result, the expression pattern shifts between WT, DA, and VA could be traced back to petal primodia initiation (stage 5, Figure S2). In zygomorphic WT, *SiCYC1s* expressions were restricted to the dorsal petal primodia (Figure 4E) and dorsal petals tips (Figure 4H). In DA peloira, *SiCYC1s* expressions were not restricted to the dorsal part of the flower, but on both dorsal and ventral petal primodia (Figure 4D), and later in all petals (dorsal, lateral, and ventral, Figure 4G). *SiCYC1s* started to express as early as the floral meristem stage both in WT and DA (Figures 4A,B). However, in VA peloria, *SiCYC1s* expression could not be detected in the floral meristem initiation stage (Figure 4C). *SiCYC1s* signals were detected on the tips of both dorsal and ventral petal primodia (Figures 4F,I), but they were obviously weaker than the signals in the wild type (Figures 4E,H).

We also found *SiCYC1s* expressions on both dorsal staminode and ventral stamen primodia in flower buds of all three cultivars (Figures 4D–I). However, this was also evident in sense controls (Figures 4J–L), so we could not be sure whether *SiCYC1s* expressed on staminode/stamens or not. The cross sections of ISH were all shown in Figure S5.

### Change of selective pressures after the duplication of *GCYC1A* and *GCYC1B*

**Figure 5:**
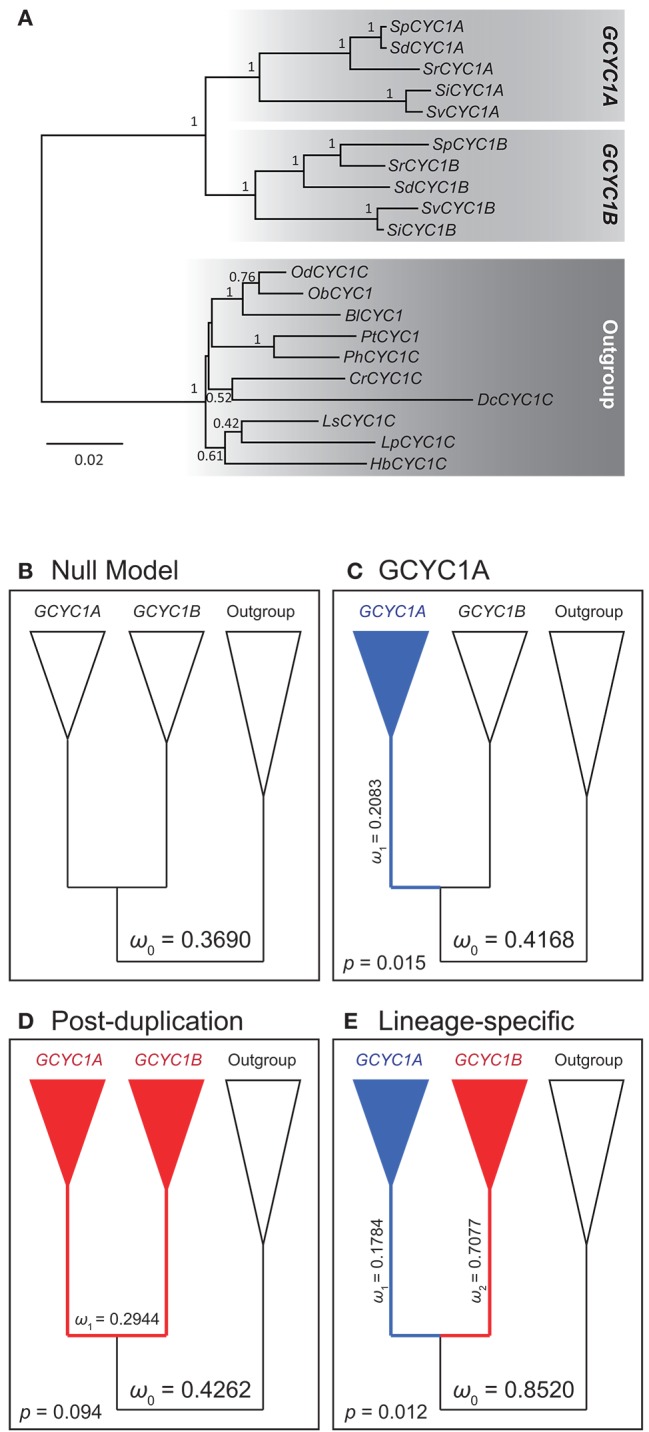
Phylogeny and Nested models (branch and branch-site) to detect selective constraint shifts between GCYC1A and GCYC1B. **(A)** Phylogeny of Generiaceae *GCYC1* by Bayesian inference was used for inference of molecular evolution by PAML. Posterior probabilities are labeled on the branches **(B–E)** Evolutionary scenarios of different models; **(B)** One-ratio null model with constant ω value; **(C)** Two-ratio model with a distinct selective pressure on *GCYC1A*; **(D)** Two-ratio model with a post-duplication alteration of selective pressure; **(E)** Lineage-specific selective pressures on *GCYC1A* and *GCYC1B* lineages estimated from clade model **C**. The ω value values follow the results in Table 1.

Our expression results suggest that *SiCYC1A* and *SiCYC1B* are expressed in different levels within the flower, implying that they are under a divergent selective regime. To test this hypothesis, we designed a series of branch models and branch site models to investigate the pattern of selection at the molecular level focusing on predating and postdating duplication events (Figure 5). Our ingroups were comprised with all available *Streptocarpus GCYC1A* and *GCYC1B* sequences (accession numbers listed in Table S4). Our analysis revealed that the evolution of *GCYC1A* (*SiCYC1A*) is obviously constrained by purifying selection but *GCYC1B* (*SiCYC1B*) showed relaxation from selection. The paml branch model results showed that *GCYC1A* clade experienced a relatively stronger purifying selection (ω_*GCYC*1*A*_ = 0.2083, Figure 5C) than background branches (ω_0_ = 0.4168) with statistic support whereas other models received no support (Table 1). When applying branch site model (clade model C), we further confirmed that almost half (*P3* = 0.4338) of the *GCYC1A* sites were under purifying selection (ω_*GCYC*1*A*_ = 0.2075). In addition, relative stronger purifying selection signal was detected after the duplication of *GCYC1A* and *GCYC1B* under post duplication scenario (ω_Post−duplication_ = 0.1472, *p* = 0.016). However, lineage-specific model is statistically most significant (*p* = 0.012) when compared to the post-duplication senario in branch-site analysis and further support that these *GCYC1A* and *GCYC1B* clades experienced divergent selection signals after their duplication. *GCYC1A* experienced purifying selection (ω_*GCYC*1*A*_ = 0.1784) whereas *GCYC1B* is relaxed from selective constraint (ω_*GCYC*1*B*_ = 0.7077) (Figure 5E, Table 1).

### Inheritance of floral symmetry in african violet

**Figure 6:**
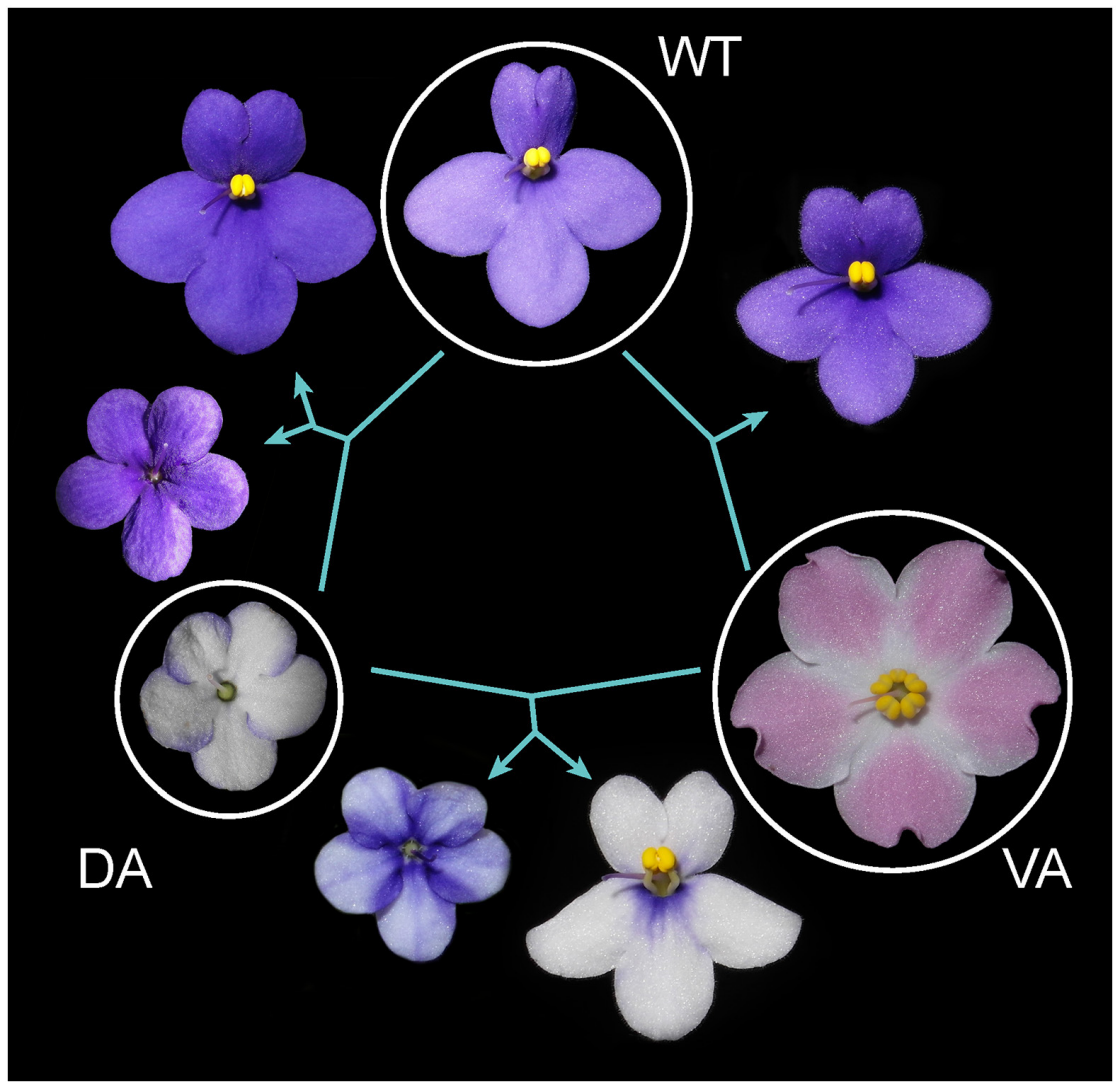
Genetic crossing designs among DA, WT, and VA. Crossings between WT and VA resulted in all F1s similar to zygomorphic WT. Crossing between two peloria VA and DA, resulted in F1s in 1:1 of zygomorphic WT and DA. Crossing between DA and WT segregated into 1:1 of DA and WT. Thus floral symmetry transitions are likely to be controlled by three alleles at a single locus in which the DA allele is dominant to WT and WT is dominant to VA. See main text for inferring their dominance hierarchy.

The genetics of floral symmetry in African violet can be revealed by genetic crosses between floral symmetry types. Crossings between WT and VA, VA and DA, also DA and WT were conducted to determine how the floral symmetry phenotypes were inherited. Selfing of WT and VA each produced phenotypes corresponding to the parent implying WT and VA are pure lines. The “No stamens” cultivar, DA however, could not be selfed owing to the lack of stamens. The crosses between WT and VA resulted in all F1 offsprings in zygomorphic WT (Figure 6). If assuming *SiCYC1s* (either *SiCYC1A* or *SiCYC1B*, or both) is the responsible locus for floral symmetry transitions, this implies that *CYC* allele of WT (*SiCYC1*^*W*^) is dominant to that of VA (*SiCYC1*^*V*^). Interestingly, the crossing between VA and DA resulted in 1:1 ratio of two phenotypes, actinomorphic DA and unexpectedly zygomorphic WT. This suggests the *SiCYC1s* allele (*SiCYC1*^*D*^) of DA is also dominant to VA (*SiCYC1*^*V*^) but DA is probably a heterozygote. This idea is further supported by the crossing between DA and WT in which the floral symmetry phenotypes of F1 hybrids were segregated into 1:1 of DA and WT, exactly corresponding to their parents (Figure 6). Thus DA (*SiCYC1*^*D*/*W*^) could be inferred as a heterozygote of *SiCYC1*^*D*^ and *SiCYC1*^*W*^, whereas WT could be referred as *SiCYC1*^*W*/*W*^. As WT is dominant to VA, pure line VA can be inferred as *SiCYC1*^*V*/*V*^. The level of dominance follows a hierarchical fashion in which *SiCYC1*^*D*^ > *SiCYC1*^*W*^ > *SiCYC1*^*V*^.

### Genetic association analysis of *SiCYC1s* to floral symmetry

To further confirm whether these *SiCYC1s* alleles are responsible for the genetic change of floral symmetry transition, genotype–phenotype associations between *SiCYC1A* and *SiCYC1B* alleles with floral symmetry phenotypes were examined among F1 hybrids of WT and DA. Among 117 F1 individuals, their phenotypes were segregated into 1:1 ratio (61 vs. 56) of WT and DA. *SiCYC1A* and *SiCYC1B* alleles of each cultivar WT, DA, and VA were cloned and sequenced to identify their segregation sites. From segregation sites sequences we recognized restriction enzymes to distinguish *SiCYC1A* and *SiCYC1B* alleles of WT and DA (Figure S6A). Here we use A to denote *SiCYC1A* and B to denote *SiCYC1B*, and thus the allelic identity of WT is *A* ^*W*/*W*^*B* ^*W*/*W*^ (WT is homozygous for both *SiCYC1A* and *SiCYC1B*) and DA is *A* ^*D*/*W*^*B* ^*D*/*W*^ (DA is heterozygous for both *SiCYC1A* and *SiCYC1B*, see results above) (Figure S6B). To genotype *SiCYC1s* alleles of each F1 individual, PCR-based RFLP was then performed. Genotype frequency test confirmed that there is no segregation distortion of these alleles among F1 hybrids (Table 2). However, when correlating genotypes to phenotypes among F1s, there is no clear association of inherited alleles (*A*^*D*/*W*^, *A* ^*W*/*W*^, *B*^*D*/*W*^, *B*^*W*/*W*^) with corresponding traits (zygomorphy, dorsalized actinomorphy) (Figure S6B).

**Table 2:** Association of *SiCYC1A* and *SiCYC1B* alleles to floral symmetry phenotypes in F1 hybrids (WT × DA).

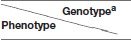	**A^D/W^B^D/W^**	**A^D/W^B^W/W^**	**A^W/W^B^D/W^**	**A^W/W^B^W/W^**	**χ^2^-value**	***P*-value**
Bilateral symmetry	14	17	10	16	–	–
Dorsalized actinomorphy	11	8	16	14	–	–
	25	25	26	30	0.6415	0.8868

*^a^ The null hypothesis of genotype frequency is 1:1:1:1. A^D/W^ denotes F1s with heterozygote SiCYC1A: A combination of one allele from DA, SiCYC1A^D^ and another allele from WT, SiCYC1A^W^. Similarly, B^D/W^denotes F1s with heterozygous SiCYC1B: A combination of one allele from DA, SiCYC1B^D^ and another allele from WT, SiCYC1B^W^. The parent DA is a heterozygote for SiCYC1A and SiCYC1B (A^D/W^B^D/W^), while parent WT is homozygous for both locus (A^W/W^B^W/W^)*.

### Expression of *SiCYC1s* alleles correlates to floral symmetry phenotypes among F1s but not to their inherited genotypes

**Figure 7:**
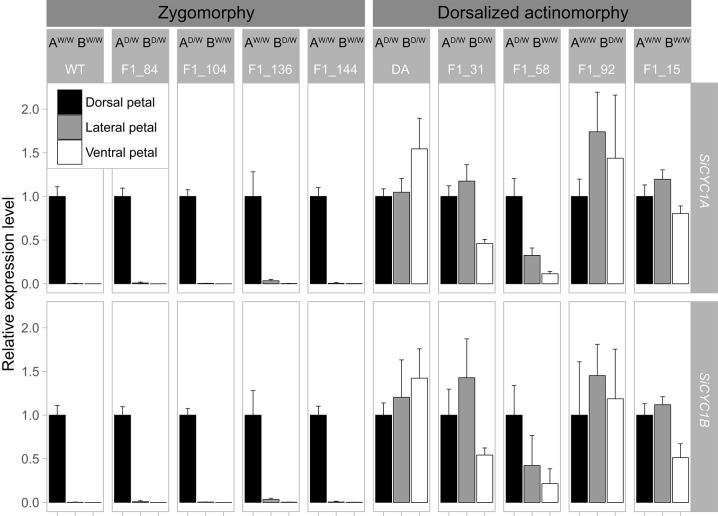
qRT-PCR expression patterns of *SiCYC1A* and *SiCYC1B* in dissected petals at stage 12 of F1 hybrids (WT × DA). This figure summarized expression patterns of *SiCYC1A* and *SiCYCB* among F1 individuals with all possible combinations of genotypes (A^D/W^B^D/W^, A^D/W^B^W/W^, A^W/W^B^D/W^, A^W/W^B^W/W^) and their phenotypes (zygomorphy, actinomorphy). For genotype abbreviation, A denotes *SiCYC1A* and B denotes *SiCYC1B*. Those F1s in zygomorphic phenotype (F1_136, F1_104, F1_84, F1_144) all share the same *SiCYC1A* and *SiCYC1B* expression pattern as their WT parent, but their inherited genotypes (allelic combinations) are in all four equally probable ways. Those F1s in dorsalized actinomorphy revealed the same phenomena in that (F1_31, F1_58, F1_92, F1_15) also share the same *SiCYC1A* and *SiCYC1B* expression pattern as their DA parent but is not confined to a certain inherited genotype. See text for the explanation. Each facet represents a biological repeat with three technical repeats (mean ± SD). The relative expression levels were normalized to *Actin* and *SiCYC1A/SiCYC1B* of the dorsal petals.

To examine whether these *SiCYC1A* and *SiCYC1B* alleles displayed distinct expression patterns correlating with floral symmetry phenotypes in F1s between WT and DA, we detected their expression level differences between dorsal, lateral, and ventral petals via quantitative RT-PCR. The expression patterns of these F1 *SiCYC1s* alleles correlate with the resulting floral symmetry phenotypes but do not necessarily follow their inherited genotype. For example, as shown in Figure 7, those four zygomorphic F1 individuals shared the same *SiCYC1A* and *SiCYC1B* expressions in the dorsal petal only, yet their *SiCYC1s* allele combinations appeared in all four equally probable ways (F1_84: *A* ^*D*/*W*^*B*^*D*/*W*^, F1_104: *A*^*D*/*W*^*B*^*W*/*W*^, F1_136: *A*^*W*/*W*^*B*^*D*/*W*^, F1_144: *A* ^*W*/*W*^*B*^*W*/*W*^). Similarly, those four dorsalized actinomorphy individuals all showed that *SiCYC1A* and *SiCYC1B* ubiquitously expressed to all petals, but their *SiCYC1s* allelic combinations were comparably the same in four equally probable ways as those in zygomorphic ones (F1_31: *A* ^*D*/*W*^*B*^*D*/*W*^, F1_58: *A*^*D*/*W*^*B*^*W*/*W*^, F1_92: *A*^*W*/*W*^*B*^*D*/*W*^, F1_15: *A* ^*W*/*W*^*B*^*W*/*W*^) (Figure 7). The cDNA products of these *SiCYC1s* alleles were further confirmed for their genotypes by sequencing. Thus, the expression shifts of these *SiCYC1A* and *SiCYC1B* alleles correlate nicely with the resulting F1 symmetry phenotype, but there lacks a specific allelic combination associating with certain symmetry phenotype.

### Heterologous expression and phenotypes of *SiCYC1A^*W*^* and *SiCYC1A^*V*^* in *Arabidopsis*

To elucidate what phenotypic effects of *SiCYC1s* are present on flower morphology, the complete coding sequences of WT allele *SiCYC1A*^*w*^ and VA peloric allele *SiCYC1A*^*V*^ were ectopically expressed into *Arabidopsis*. We did not further examine the over expression phenotype of *SiCYC1A*^*D*^ since the overexpression phenotypes of *SiCYC1A*^*w*^ and *SiCYC1A*^*V*^ in *Arabidopsis* were almost identical, and the allelic difference between *SiCYC1A*^*D*^ to *SiCYC1A*^*w*^ and *SiCYC1A*^*V*^ is in just one non-synonymous amino acid substitution. In particular, this single non-synonymous amino acid substitution (position 86 in alignment of Figure 2A) in *SiCYC1A*^*D*^ is not on important domains such as TCP, ECE, or R.

**Figure 8:**
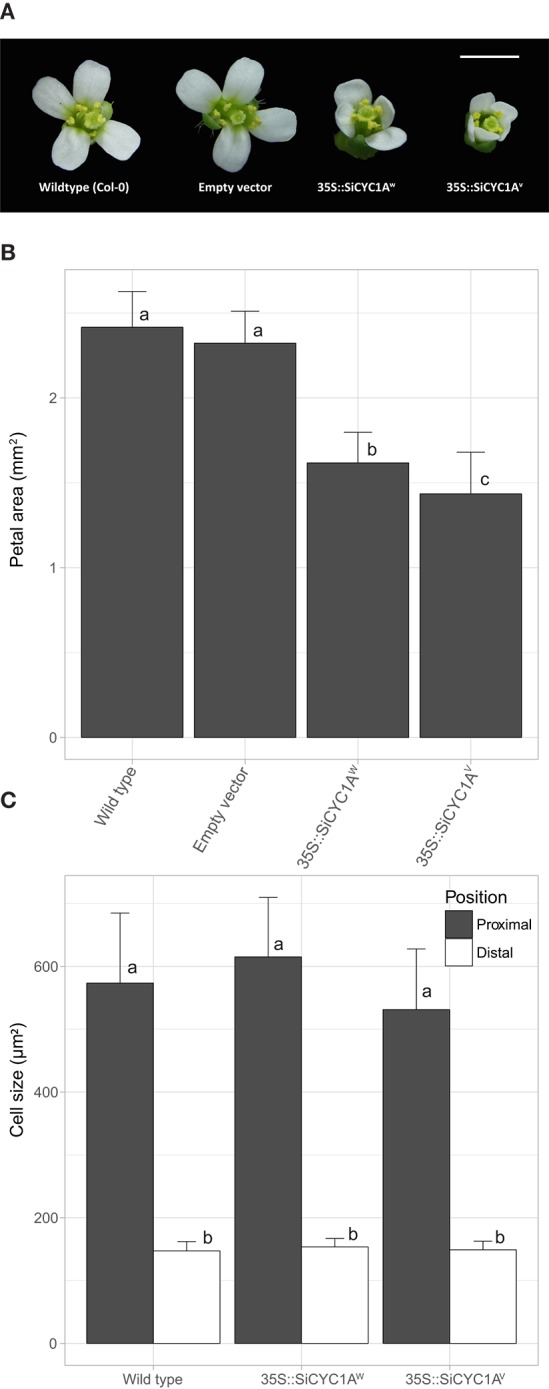
Flower morphology of Arabidopsis T2 transgenic plants. **(A)** Constitutive expression of different alleles of *SiCYC1A* in *Arabidopsis* represses petal size. Flower morphology of T_2_ transgenic plants were measured at fully open stage. Scale bar = 1.5 mm. **(B)** Petal size (mean ± SD) decreases in *SiCYC1A* overexpression T_2_ transgenic plants [*F*_(3, 150)_ = 150.6, *p* < 0.001]. **(C)** Cell size (mean ± SD) in petals of T_2_ transgenic plants are not different to the wild type. Thus *SiCYC1A* has effects on reducing cell proliferation to repress petal size. The significance was analyzed by ANOVA test and grouped by Turkey *post hoc* test (significant level = 0.05). See Figure S8 for cell morphology in scanning electronic microscope.

Overexpression of *SiCYC1As* in *Arabidopsis* affects the flower size and leaf growth with smaller petals and leaf recurving (Figure 8, Figure S7). The *SiCYC1A*^*W*^ and *SiCYC1A*^*V*^ T2 transgenic plants generally produced smaller and narrower petals than those in WT *Arabidopsis* and empty vector control. To further understand whether the reduction of the petal size by *SiCYC1A*^*W*^ and *SiCYC1A*^*V*^ is caused by reduction of cell proliferation or cell expansion, petal size and cell size of T2 plants were measured by the following procedures as stated above. The measurements were averaged by 20 petals from five flowers per individual, three individuals of each construct. The average size of *Arabidopsis* wild-type petals and empty vector control was measured to be 2.4 mm^2^ (±0.21) and 2.3 mm^2^ (±0.18), respectively. However, petals of T_2_ plants of *SiCYC1A*^*W*^ and *SiCYC1A*^*V*^ had a reduced petal size of 1.6 to 1.4 mm^2^ (±0.17~0.26) therefore were 1.5-fold to 2.0-fold smaller than *Arabidopsis* WT petals (Figure 8B) [ANOVA test, *F*_(3, 150)_ = 150.6, *p* < 0.001].

The cell sizes (including distal and proximal regions) differences between each construct were further compared (Figure 8C). Both in *SiCYC1A*^*W*^ and *SiCYC1A*^*V*^ T_2_ transgenic plants, the cell size in the proximal petal region (615.10 ± 94.55 μm, 531.30 ± 96.41 μm, respectively) showed no difference to that in the wild type (573.54 ± 111.33 μm). Similarly, no cell size difference could be found in the distal petal region among T_2_ plants and wild type. Together, these suggest that the reduction of petal size by *SiCYC1A*^*W*^and *SiCYC1A*^*V*^ in *Arabidopsis* T2 transgenic plants is caused by reduced cell proliferation rather than cell size changes, similar to that in African violet. This is evident from SEM pictures that there is no conspicuous morphological difference on petal epidermal cell among wildtype, *35S::SiCYC1A*^*W*^, and *35::SiCYC1A*^*V*^ transgenic plants. The distal side petal cells in transgenic T2 looks normal as conical cells and at the proximal side cells are long and columnar, resembling those of the wild type (Figure S8). For leaf morphology, the *SiCYC1A*^*W*^ and *SiCYC1A*^*V*^ T_2_ transgenic plants had thicker and more curly leaves than that in wild type and empty vector control (Figure S7). And this effect was more severe in *SiCYC1A*^*V*^ T_2_ transgenic plants than it was in *SiCYC1A*^*W*^ T_2_ transgenic plants.

## Discussion

### Divergent expression shifts of *SiCYC1s* associate with reversions to actinomorphy

We have demonstrated that *SiCYC1*s (*SiCYC1A* and *SiCYC1B*), similar to *CYC* and *DICH* in snapdragon, have a dorsal petal-specific expression pattern in floral meristems (*RNA in-situ*) and late petal development stages (qRT-PCR) of WT (Figures 3, 4). The expression of *SiCYC1*s correlates to smaller dorsal petal size observed in WT. The extended *SiCYC1*s expression to all petals in DA also correlates with the fact that all five petals are small in size and all five stamens are aborted. The much reduced *SiCYC1*s expression in VA, although still dorsal specific, correlates positively with enlarged dorsal petals similar to the size of lateral and ventral petals. Therefore, the shifts of *SiCYC1*s expression correlate to floral symmetry transitions in both DA and VA.

From our study, it is unique that the petal homeotic transformation into dorsal and ventral identity both evolved in African violet peloria. In Gesneriaceae, dorsalized actinomorphy has been reported in *Tengia* and occasionally in *Petrocosmea* hybrids (Pang et al., 2010; Yang et al., 2015). On the other hand, ventralized actinomorphy attributable to mutated *CYC* or cessation of expression of *CYC* in late petal development stage has been reported in *Sinningia speciosa* and *Bournea* (Zhou et al., 2008; Hsu et al., 2015, 2017). Other than Gesneriaceae, the evolutionary reversals to dorsalized actinomorphy attributable to the extension of *CYC* into all petals have been reported in *Cadia* (Leguminosae), parallel Malpighiaceae lineages (Citerne et al., 2006; Zhang et al., 2010, 2012, 2013). But reversals to actinomorphy owing to loss of asymmetrical *CYC* expression or transient expression only are more frequently observed such asin *Tradescantia* (Commelinaceae, monocot), in *Plantago lanceolate* (Plantaginaceae, eudicots), in pollinator shifted Malpighiaceae lineages and in *Arabidopsis* (Cubas et al., 2001; Reardon et al., 2009; Preston et al., 2011; Preston and Hileman, 2012; Zhang et al., 2013).

It is worth mentioning that the shifts of *CYC* expression have also been demonstrated to correlate with patterns of stamen arrest. In *Mohavea confertiflora* (Plantaginaceae), expansion of *CYC* expression into lateral stamen primordia correlates with the abortion into sterile staminodes (Hileman et al., 2003). Our finding clearly indicates that *SiCYC1A / 1B* are still weakly expressed in the dorsal part of the ventralized actinomorphic flower (VA) and this may explain why the VA peloria shows some residual asymmetry in having the dorsal stamen slightly reduced in size when compared to lateral and ventral ones (Figures 1C,F, Figure S2AA). On the other hand, ubiquitous expression of *CYC* in the entire flower bud, as in DA of African violet, may retard all stamen development (Figure S2Y). However, the function of *CYC* may not be necessary to link to petal growth and stamen development in all cases. In dorsalized actinomorphy of *Tengia* and *Cadia*, the whole flower expression of *CYC* apparently does not contribute to stamen arrest (Citerne et al., 2006; Pang et al., 2010). Therefore, the regulation shifts of *CYC* expression correlating to floral symmetry transition, by means of controlling petal and stamen development in different parts of the flower, could be far more complicated and diverse than one could imagine.

### *SiCYC1s* gene duplication resulted in divergence of gene expression and divergent selection

Gene duplication is usually accompanied by increased gene expression diversity. Our results clearly indicate that *SiCYC1A* acts as the major expressed copy whereas *SiCYC1B* as the weakly expressed one in all studied floral tissues (Figure 3). Given the evidence that *SiCYC1A* and *SiCYC1B* are apparently recently duplicated paralogues and their amino acid sequence similarity is as high as 87% (Figure 2, see also Wang et al., 2004a), *SiCYC1A* and *SiCYC1B* thus act redundantly. This is reminiscent of *Antirrhinum* in that *CYC* expressed stronger when compared to weaker *DICH* expression in the dorsal part of the flower (Luo et al., 1996, 1999).

Consistent with previous findings (Wang et al., 2004a), an ancient *GCYC* duplication event (*GCYC1* vs. *GCYC2*) was also uncovered from our analysis (Figure 2B). This duplication seems to predate the origin of all Gesneriaceae species owing to the fact that every species contains both *GCYC1* and *GCYC2* copies including our African violet cultivars. Certain old world Gesneriaceae, particularly Didymocarpoideae species, also contain another subfamily level duplication of *GCYC1* into *GCYC1C* and *GCYC1D* (Wang et al., 2004a), which is also revealed in our *GCYC* phylogeny tree. Whether or not these various levels of *GCYC* duplication resulted in a diversified expression pattern remains to be investigated.

Gene duplication may lead to the conservation of major function in one copy but allow a variation in the other copy. Analysis of our *GCYC1A* / *1B* gene dataset suggests a purifying selection constraint on the major high-expressed copy, *GCYC1A* (*SiCYC1A*), whereas the weakly expressed *GCYC1B* (*SiCYC1B*) showed relaxation of purifying selection since their duplication, in a lineage-specific manner (Figure 5E). Hileman and Baum (2003) also detected a relaxed purifying selection along the *DICH* linage whereas *CYC* lineage remains under strong genetic constraint. They hypothesized that duplication of *CYC*/*DICH* in *Antirrhinum* occurred in three stages: (1) duplication; (2) relaxed selection in one of the duplicated genes allowing to acquire sub or new function; and (3) purifying selection to stabilize both duplicated genes to maintain evolved functions.

Frequent *CYC* duplications followed by their diverse expression patterns have been proposed as a driving force for their functional divergence (Zhong and Kellogg, 2015). In *Lupinus*, positive selection acting at few *LEGCYC1B* sites were correlated with petal size changes in *L. densiflorus* (Ree et al., 2004), suggesting neo-functionalization of this *CYC* duplicate. In *Helianthus*, divergent expression patterns of *CYC* duplicates were correlated to residues in the conserved domains under positive selection following gene duplication (Chapman et al., 2008). Bello et al. (2017) has uncovered that each *CYC/TB1* duplicate has evolved either purifying or episodic positive selection among Asteraceae species thus allowing retention of multiple copies of *CYC* for generating flower shape diversity. To conclude, the rapid evolution through gene duplications and sub-functionalization among diversified copies could be a common regime for *CYC* evolution.

### Genetic analysis reveals trans-acting factors of *SiCYC1s* required for floral symmetry transition

Cis-regulatory changes, trans-regulatory changes, and a combination of both have been shown to contribute to the expression differentiation between *CYC* duplicates in Gesneriaceae species (Yang et al., 2012, 2015). *CYCs* in Generiaceae species can positively auto-regulate themselves (cis regulatory effect) and cross-regulate each other (trans regulatory effect) (Yang et al., 2012). Thus the shifts of *SiCYC1s* expression and the resulting floral symmetry transitions in African violet could be attributable to the difference in efficiency of positive auto- and cross- feedback for maintenance of expression between *SiCYC1s* alleles. For example, the low expression level of *SiCYC1A*^*V*^ in VA flower when compared to higher *SiCYC1A*^*W*^ in WT could be attributable to a mutation on auto- or cross- regulatory feedback loop of *SiCYC1A*^*V*^ thus resulting in poor maintenance of its expression level.

From supposed dominance or recessiveness of allelic relationship inferred from our crossing design results, we expect that F1s of WT x DA with dorsalized actinomorphy phenotype should have inherited at least one DA allele (*SiCYC1A*^*D*^ or *SiCYC1B*^*D*^) from the DA parent (Figure 6, Figure S6B). To our surprise, from F1s genotyping results, there is an equal association of DA or WT allele (*SiCYC1A*^*W*^ or *SiCYC1B*^*W*^) to dorsalized actinomorphy (Table 2). The same conclusion also applies to those F1s with zygomorphic phenotype. Even though there is no clear association of certain *SiCYC1s* alleles to particular floral symmetry phenotypes in F1s, expression patterns of these alleles, however, match perfectly with their resulting phenotypes (Figure 7). Allelic sequences of *SiCYC1s* are highly similar to each other among cultivars (< 1% divergence at amino acid level) and thus unlikely to evolve into new phenotypic functions. Adding to the fact that ectopically expressing CDS of either *SiCYC1A*^*W*^ or *SiCYC1A*^*V*^ gives the same phenotypic effect on *Arabidopsis* petal morphology, the function of *SiCYC1s* alleles from different African violet cultivars is perhaps identical. Thus, the shifts of *SiCYC1s* expression and the resulting floral symmetry transitions are more likely controlled by trans-acting factors upstream of *SiCYC1s* rather than their own cis-elements.

Several recent studies also suggest the possibility that other dorsiventral asymmetrically expressed genes rather than *CYC* could be the actual controlling factors of zygomorphy. This is evident in *Aristolochia*, Proteaceae, and Fumarioideae species where *CYC* differential expression along the dorsiventral axis in flower was observed only after the perianth zygomorphy had already been established (Damerval et al., 2013; Horn et al., 2015; Citerne et al., 2017). Trans-acting (upstream) factors other than *CYC* could therefore be the other possibility accounting for the shifts of *CYC* expression by either promoting or repressing the transcription of *CYC* in certain parts of the flower.

Although there are no reports on what *CYC* trans-acting (upstream) regulators should be, B class genes which specify petal and stamen identity were possible candidates. Only few studies ever managed to test this hypothesis yet their interaction remains unclear. In *Antirrhinum*, B class gene (*DEFICIENS*) activity is required for maintenance of *CYC* in petal whorl (Clark and Coen, 2002). Preston and Hileman (2012) when compared to *B* class (*DEF* and *GLO*) and *CYC* (*TB1*) expressions in zygomorphic *Commelina* and actinomorphic *Tradescantia* species (Commelinaceae). They found *CYC* expressed more in ventral parts (inner tepals and stamens) of the flowers but B class exclusively more in dorsal parts. B class genes especially *AP3-2* and *AGL6-2* have been found in orchids involving lip formation since their expressions are concentrated in the flower's ventral part, namely the lip (Pan et al., 2011; Hsu et al., 2015). This asymmetrical expression pattern thus helps to create zygomorphy in *Oncidium* and *Phalenopsis* orchids. Further works on whether B-class genes and *CYC* interact with each other shall resolve their regulation relationship.

### Epigenetic regulation of *SiCYC1s* may explain floral symmetry transition in african violet

DNA epigenetic mutation on *CYC*, although rarely reported, is also a plausible mechanism to account for the shifts of *CYC* expression in generating floral symmetry transition. *CYC* was transcriptionally silent and found to be extensively methylated in peloric mutant but not in the zygomorphic wildtype of *Linaria vulgaris* (Cubas et al., 1999b). Flowers from peloria in *L. vulgaris* show somatic instability and later develop reversely to zygomorphy, correlating with the demethylation and restoration of *CYC* expression. Epigenetic regulation is more likely the reason for the shifts of *SiCYC1s* expression in African violet since we also observed that F1 hybrids with dorsalized actinomorphy phenotypically reverts into zygomorphic WT within the same inflorescence (Figure S9). African violet is not alone. Among hybrids of two zygomorphic *Petrocosmea* species, individuals reversed to dorsalized actinomorphy with all stamens aborted can sometimes be observed (Yang et al., 2015). Therefore, the changes of epigenetic regulation on *CYC* could be a more widespread scenario but was previously neglected. Although not yet reported in *CYC*, a 4.9 fold increase in histone acetylation level has been reported for B class gene (PeMADS4) in the lip of *Phalaenopsis* orchid, correlating to its high expression in lip specifically (Hsu et al., 2014). Further works on checking the DNA methylations and histone acetylation levels among *SiCYC1s* alleles from flower parts between different cultivars are needed to prove this.

### *SiCYC1a* function to retard petal growth via reduced cell proliferation and higher expression

*CYC* duplicates appear to evolve into a diverse phenotypic effect on petal morphology. In Gesneriaceae species, including our results, the high expression copy, *SiCYC1A*, appears to have a major role in restricting petal size of dorsal petals in African violet and in petals of transformed *Arabidopsis*. Similarly, two *Petrocosmea* species have their *CYC1C* and *CYC1D* expressing restrictively in dorsal petals (Yang et al., 2015). But both copies showed much higher expression in *one* species, *P. glabristoma*, in correlating to its smaller dorsal petal size, whereas a weaker expression in another species, *P. sinensis*, was associated to its larger dorsal petal size. Through genetic association studies, *CYC* mutation has been associated with the enlargement of the entire flower and petal in artificially selected VA of *Sinningia speciosa* (Hsu et al., 2015). From corolla 3D geometric morphometric analysis, *SsCYC* has been demonstrated as associating with the outward curvature of dorsal petals and petal size, again, a strong support that *CYC* affects diverse parts of petal morphology (Wang et al., 2015; Hsu et al., 2017).

The function of *SiCYC1A* in retarding African violet petal growth was further confirmed by their phenotypes in *Arabidopsis* transformants with apparently reduced petal size (Figure 8). This petal retarding effect is congruent with the ectopic expression phenotypes of *CYC1C* from *Primulina heterotricha* and *TCP1* genes from *Iberis amara* in *Arabidopsis* (Busch and Zachgo, 2007; Yang et al., 2012). In contrast with that, however, *Antirrhinum CYC* promotes petal growth in *Arabidopsis* (Costa et al., 2005). This demonstrates that *CYC* genes in diverse lineages have evolved contrasting function on petal growth although their major roles in floral symmetry remain unchanged.

While looking in detail on whether the *CYC* effects on petal growth was achieved by regulating cell proliferation or expansion, different conclusions were drawn. From petal epidermal picture in African violet cultivars and in *Arabidopsis* transformants, it appears that the effect of *SiCYC1A* on petal size is attributable to reduced cell proliferation (cell division) rather than reduced cell growth (Figures S4, S8). This is again a different observation when compared to the effect of *CYC1C* from *Primulina heterotricha* and *CYC* from *Antirrhinum CYC*, in which they retard cell growth (reduced size of cells) but not cell proliferation (Costa et al., 2005; Yang et al., 2012). These facts imply that the regulation of *CYC* on petal morphology is far more complicated than we thought and they may evolve different modified functions independently in various flowering plant lineages.

### Homologs of *RADIALIS* and *DIVARICATA* isolated from african violet but their expressions showed no dorsiventral asymmetric patterns

In *Antirrhinum CYC* regulates a downstream MYB-like protein *RADIALIS* (*RAD*) to establish zygomorphy (Galego and Almeida, 2002; Corley et al., 2005; Costa et al., 2005). *CYC* protein directly binds to the *RAD* promoter in the dorsal part of the flower, allowing RAD protein to antagonistically compete with another MYB gene family *DIVARICATA* (DIV) protein for other two downstream MYB-like proteins to prevent DIV activity in dorsal regions (Costa et al., 2005; Raimundo et al., 2013; Hileman, 2014b). *rad* ^−^mutants of *Antirrhinum* have enhanced ventral identity but retained the dorsal identity (Carpenter and Coen, 1990). A ventralized peloric phenotype could therefore also result from a knock-out *RAD* mutation.

We also isolated putative *RAD* and *DIV* homologs from WT, DA and VA (Figures S10, S11, detailed methods please see Supplementary Materials). The (q)RT-PCR expression patterns of *RAD* (*SiRAD1* and *SiRAD2*) and *DIV* (*SiDIV1A* and *SiDIV1B*) (Figures S12, S13), however, were not correlated with *SiCYC1s* expression shifts in these cultivars. One could argue that our PCR cloning process may not isolate all possible homologs of *RAD* and *DIV*, given they belong to part of multigenic *MYB* families. It is therefore not clear whether *RAD* and *DIV* are associated with floral symmetry transitions in *S. ionantha*. Detailed results of this section were provided in the Supplementary Material and the findings were briefly discussed there.

## Author contributions

C-NW designed the research and carried out the main writing. The experiments were conceived and conducted by H-JH, C-WH, and W-HK. Experimental strategies were designed by Z-JP and C-NW. Gene selection analysis was performed and interpreted by K-TH, J-YL, and C-NW. All authors prepared and commented on the manuscript.

### Conflict of interest statement

The authors declare that the research was conducted in the absence of any commercial or financial relationships that could be construed as a potential conflict of interest.
