# Erratum: Genetic Analysis of Floral Symmetry Transition in African Violet Suggests the Involvement of Trans-acting Factor for *CYCLOIDEA* Expression Shifts

**DOI:** 10.3389/fpls.2018.01707

**Published:** 2018-11-23

**Authors:** Frontiers Production Office

**Affiliations:** Frontiers Media SA, Lausanne, Switzerland

**Keywords:** *CYCLOIDEA*, *Saintpaulia ionantha*, zygomorphy, actinomorphy, *RADIALIS*, genotype–phenotype association, gene duplication, bilateral symmetry

**Reason for Erratum:**

Due to a typesetting error, the terms CYCs and SiCYC were used in the Abstract. The correct terms should be SiCYC1s and SiCYC1A respectively.

In the Introduction, paragraph 1 “small sizes” should be “small-sized.”

In the caption for Figure 2 the second mention of SiCYC1A should be SiCYC1B.

In Results, Cell Proliferation but Not Cell Growth Cause Dorsiventral Petal Size Difference in African Violet, paragraph 2 “Petal area was” should be “Both petal area and cell size were”.

In Results, Expression Shifts of SiCYC1s Correlates to Developmental Reversals to Actinomorphy in DA and VA Peloria, GCYC1 should be SiCYC1s.

In Results, subsections Inheritance of Floral Symmetry in African Violet, and Genetic Association Analysis of SiCYC to Floral Symmetry, SiCYC and CYC should be SiCYC1s.

In Discussion, the following subsection headers are incorrect. The first should be SiCYC1s instead of CYC and the second be SiCYC1s instead of SiCYC.

- Divergent Expression Shifts of CYC Associate With Reversions to Actinomorphy
- SiCYC Gene Duplication Resulted in Divergence of Gene Expression and Divergent Selection.

The publisher apologizes for this mistake. The original article has been updated.

