# Supplementary material for: Genetic Analysis of Floral Symmetry Transition in African Violet Suggests the Involvement of Trans-acting Factor for CYCLOIDEA Expression Shifts
Source: Front Plant Sci. 2018 Aug 15;9:1008. doi: 10.3389/fpls.2018.01008 (PMC6104639; doi:10.3389/fpls.2018.01008)
Supplement: Supplementary file 1 [file Data_Sheet_1.docx]

Supplementary Material

Genetic analysis of floral symmetry transition in African violet suggest involvement of trans-acting factor for *CYCLOIDEA* expression shifts

Hui-Ju Hsu^1†^, Cheng-Wen He^1†^, Wen-Hsi Kuo^1†^, Kuan-Ting Hsin^1†^, Jing-Yi Lu^1^, Zhao-Jun Pan^2†^ and Chun-Neng Wang^1,2*^

* Correspondence: Chun-Neng Wang: leafy@ntu.edu.tw

# Supplementary Materials and Methods

## Cytometric ploidy analysis

To confirm whether the ploidy level is different between African violet cultivars WT, DA, and VA, tissue DNA content were measured by the flow cytometry based on Kuo et al. (2017) protocols. The Beckman buffer (1% Triton X-100, 50mM sodium sulfite, 50mM pH 7.5 Tris-HCl, 0.1 % β-mercaptoethanol, 4% PVPP) was freshly prepared and pre-cooled on ice. The leaf tissue were chopped with razors in 1.5 ml of Backmen Buffer, and the chopped tissues were placed on ice and ﬁltered through 30-um nylon meshes (Partec, Munster, Germany). The sample was subsequently stained with a 1 : 50 volume of propidium iodide (PI) solution (2.04 g/ml) and incubated at 4°C in the dark for 1 h. Analyses of the sample was performed on a BD FACSCan system (BD Biosciences, Franklin Lake, NJ, USA).

**Isolation of *RAD* and *DIV* homologs**

Part of the *SiRAD1* and *SiRAD2* were first isolated with primers AKE-F1 (5’-GGAATAAACCTGGAATCTTCAGTTACC-3’) and PFP-R1 (5’-TCACTTGTTTCCAGTGGTCCAGTAGTTGGGAAAGG-3’) designed at conserved MYB domain from aligned available *RADIALIS* sequences (*Antirrinum majus*, *Bournea* and *Lycopersicon*) in Genbank (**Figure S10**, **Table S2**). After these partial sequences obtained and cloned to identify as *SiRAD1* and *SiRAD2*, genome walking toward 5’end and 3’RACE techniques (as above) were applied to get their full length sequences.

Using similar strategy, partial sequences of the *SiDIV1A* and *SiDIV1B* were first isolated with primers DVM-F1 (5’-GTGCCGGGGAAAACCGTGGTGGATGT-3’) and HDI-R1 (5’-GAGGCYGGGCTGATCCCAGTRCCTGG-3’) designed at conserved MYB domain from aligned available *DIVARICATA* (*DIV*) *and DIVARICATA-like* (*DVL*) sequences (*Antirrinum majus*, *Bournea* and *Lycopersicon*) in Genbank (**Figure S11**, **Table S2**). After these partial sequences obtained and cloned for the identity of *SiDIV1A* and *SiDIV1B*, 5’end genome walking and 3’RACE were applied to get their full length sequences.

## Phylogenetic analysis of *RADs* and *DIVs*

In order to infer the phylogenetic relationship of *SiRADs* and *SiDIVs* duplicates, maximum likelihood (ML) and Bayesian inference (BI) trees were reconstructed as the methods of phylogenetic analysis of *CYCs* in main materials and methods. The nucleotide sequences of available Genbank Gesneriaceae *RAD* and *DIV* sequences and those from *Antirrhinum* were aligned with *SiRADs* and *SiDIVs*, respectively, based on their amino acid sequences using MAFFT (<https://mafft.cbrc.jp/alignment/server/>) and manually adjusted.

# Supplementary Results

## The phylogeny of isolated *RAD* and *DIV* homologs and duplications identified

To identify putative genes involved in floral symmetry in African violet, two of *RAD*-like genes (*SiRAD1*, *SiRAD2*), and two of *DIV*-like genes (*SiDIV1A*, *SiDIV2B*) were isolated in WT, DA, and VA (**Figure S10A**, **S11A**). Maximum likelihood (ML) and Bayesian inference (BI) trees were reconstruction to examine the homologies of putative *RAD*-like genes, and *DIV*-like genes (**Figure S10B**, **S11B**). Sequences from actinomorphic peloric cultivars, DA and VA, were not found to have any frameshift mutations (all indels were in multiple of three nucleotides) or stop codons.

*SiRAD1* and *SiRAD2* in WT shared 73.0% and 71.4% amino acid identity with *RAD* from *Antirrhinum majus*, respectively. The divergence between *SiRAD1* and *SiRAD2* was 23.9% at the amino acid level and 26.9% at the nucleotide level in WT. The full length ORF of *SiRAD1* (246 bps) and *SiRAD2* (261 bps) were conserved in length except some SNP sites exists among three African violet cultivars. Sequences of *SiRAD1* were identical among three cultivars, and sequences of *SiRAD2* shared 90.9% identity between WT and VA at the amino acid level*.* Both *SiRAD1* and *SiRAD2* have one conserved 56aa MYB domain (**Figure S10A**). Phylogenetic analyses showed that *SiRAD1* and *SiRAD2* formed two distinct clades with sufficient support. With *Lycopersicon esculentum LyeFSM1* as outgroup, *SiRAD1* formed one monophyletic clade with Didymocarpoid Generiaceae species, such as *CrRAD1* of *Conandron ramondioides*, and *Antirrhinum majus* (*AmRAD*). While *SiRAD2* formed another monophyletic clade with *CrRAD2* of *Conandron ramondioides* and *RAD*-like gene (*BlRAD*) of *Bournea leiophylla* according to Maximum likelihood (ML) and Bayesian inference (BI) trees (**Figure S10B**).

*SiDIV1A* and *SiDIV1B* in WT shared 68.8 % and 65.7% amino acid identity with *DIV* from *Antirrhinum majus*, respectively. The divergence between *SiDIV1A* and *SiDIV1B*  was 13.6% at the amino acid level and 10.6% at the nucleotide level in WT. The full length ORF of *SiDIV1A* (900 bps) and *SiDIV1B* (903 bps) were conserved in length except some SNP sites exists among three African violet cultivars. Percent sequence divergence were low between *SiDIV1A* (1%) and *SiDIV1B* (1%) at the amino acid level. Conserved SANT domain and MYB-specific motif “SHAQKY” were found in both *SiDIV1A* and *SiDIV1B* (**Figure S11A**). Phylogenetic trees reconstructed from nucleotide showed that *SiDIV1A* and *SiDIV1B* formed a monophyletic clades with *BlDIV1* and *BlDIV2* of *Bournea leiophylla* with *Antirrhinum majus AmDIV* and *AmDVL* (*DIV*-like gene) as outgroup (**Figure S11B**)*.*

# Supplementary References

Baxter, C.E.L., Costa, M.M.R., and Coen, E.S. (2007). Diversification and co-option of RAD-like genes in the evolution of floral asymmetry. *Plant J* 52(1), 105-113. doi:10.1111/j.1365-313X.2007.03222.x

Boyden, G.S., Donoghue, M.J., and Howarth, D.G. (2012). Duplications and Expression of RADIALIS-Like Genes in Dipsacales. *International Journal of Plant Sciences* 173(9), 971-983. doi:10.1086/667626

Costa, M.M., Fox, S., Hanna, A.I., Baxter, C., and Coen, E. (2005). Evolution of regulatory interactions controlling floral asymmetry. *Development* 132(22), 5093-5101. doi:10.1242/dev.02085

Garces, H.M., Spencer, V.M., and Kim, M. (2016). Control of Floret Symmetry by RAY3, SvDIV1B, and SvRAD in the Capitulum of Senecio vulgaris. *Plant Physiol* 171(3), 2055-2068. doi:10.1104/pp.16.00395

Kuo, L.Y., Huang, Y.J., Chang, J., Chiou, W.L., and Huang, Y.M. (2017). Evaluating the spore genome sizes of ferns and lycophytes: a flow cytometry approach. *New Phytol* 213(4), 1974-1983. doi:10.1111/nph.14291

Möller, M., Clokie M., Cubas, P., and Cronk, Q.C.B. (1999) “Integrating molecular and developmental genetics: a Gesneriaceae case study” in Molecular Systematics and Plant Evolution, ed. P.M. Hollingsworth, R.M. Bateman, R.J. Gornall (London, Taylor and Francis), 375-402

Preston, J.C., Kost, M.A., and Hileman, L.C. (2009). Conservation and diversification of the symmetry developmental program among close relatives of snapdragon with divergent floral morphologies. *New Phytol* 182(3), 751-762. doi:10.1111/j.1469-8137.2009.02794.x

Troutt, A.B., McHeyzer-Williams, M.G., Pulendran, B., Nossal, G,J. (1992). Ligation-anchored PCR: a simple amplification technique with singlesided specificity. *Proc Natl Acad SciUSA* 89:9823–9825. doi: 10.1073/pnas.89.20.9823

Wang, C.N., Moller, M., and Cronk, Q.C.B. (2004). Phylogenetic position of Titanotrichum oldhamii (Gesneriaceae) inferred from four different gene regions. *Systematic Botany* 29(2), 407-418. doi 10.1600/036364404774195593

Zhou, X.R., Wang, Y.Z., Smith, J.F., and Chen, R. (2008). Altered expression patterns of TCP and MYB genes relating to the floral developmental transition from initial zygomorphy to actinomorphy in Bournea (Gesneriaceae). *New Phytol* 178(3), 532-543. doi:10.1111/j.1469-8137.2008.02384.x

# Supplementary Figure legends

**FIGURE S1| Ploidy levels checking between WT, DA, and VA via flow cytometry.** The G1 peak of WT nuclear DNA content is positioned at channel 186.98 (A), the DA is 212.78 (B), and the G1 peak of the VA sample appear in the channel 202.93 (C). Because all G1 peak of three cultivars are relatively in same position, all cultivars, WT, DA and VA are estimated to be in same ploidy level.

**FIGURE S2| Floral development process in African violet before stage 7 under scanning electron microscope.** Stage definitions follow **Table S1**. Bars =50 μm

**FIGURE S3|** **Photos of floral developmental from stage 8 to fully open (stage 16) in African violet.** (A) DA. (B) WT. (C) VA. Stage definitions follow **Table S1**. Bars =1 cm.

**FIGURE S4| Comparison of epidermal cell morphology between dorsal, lateral and ventral petals in WT, DA, and VA via scanning electron microscope.** Epidermal cell morphology on distal and proximal region of dorsal, lateral, and ventral petal in WT, DA, and VA. Bars =200 um.

**FIGURE S5| In situ hybridization of *SiCYC1A* in flower buds (cross sections) of African violet.** Patterns of RNA *in situ* hybridization of *GCYC1* hybridized with antisense probes of *SiCYC1A* in flower buds along horizontal axis sections of DA, WT, and VA. Dorsal petals, lateral petals and ventral petals are denoted by dp, lp, vp, respectively. Stamen and staminodes are denoted by st and *. Sepal is denoted by se. Abaxial side of flower bud is at bottom (base) of each photo. Bars =100 μm.

**FIGURE S6| Association of floral symmetry phenotypes and *SiCYC1s* genotypes among selected F1 individuals.** (A) PCR-RFLP banding patterns for discrimination of *SiCYC1A* and *SiCYC1B* allelic combinations in DA and WT parents. (B) Top row, zygomorphic F1s with all 4 possible allelic combinations of *SiCYC1A* and *SiCYC1B*. Bottom row, dorsalized actinomorphic F1s with all possible allelic combinations of *SiCYC1A* and *SiCYC1B*.

**FIGURE S7| Phenotypic analysis of transgenic Arabidopsis T_2_ plants.** (A) Adaxial surface and (B) Abaxial surface of mature leaves, bar= 2 cm. (C) Individual size, bar= 5 cm. From left to right: wild type (Col-0), empty vector transgenic plant, *35S::SiCYC1A^W^* transgenic plant, and *35S::SiCYC1A^V^* transgenic plant.

**FIGURE S8| Petal cell morphology (Adaxial side) of transgenic Arabidopsis T_2_ plants.** (A) one petal with distal side and proximal side indicated. bar= 400 um. (B-G) distal side (top row) and proximal side (bottom row) of the adaxial petal, bar= 50 um. From left to right: wild type, *35S::SiCYC1A^W^* and *35S::SiCYC1A^V^*.

**FIGURE S9| Floral symmetry transition within the same inflorescence implying *CYC* in African violet subject to epigenetic control.** No.140 of F1 hybrid (WT × DA) has actinomorphic DA flowers, but in its inflorescences certain first flowers reversed to zygomorphic WT (white arrow).

**FIGURE S10| The phylogeny of *RAD* including *SiRADs* from selected Gesneriaceae species.** (A) Alignment of *SiRAD1* and *SiRAD2* with *AmRAD* (snapdragon). MYB domains are indicated. (B) The tree with tomato LeFSM1 as outgroup implies that Gesneriaceae *RAD* homologs appear to duplicate into *RAD1* and *RAD2* clades.

**FIGURE S11| The phylogeny of *DIV* including *SiDIV1s* from selected Gesneriaceae species.** (A) Alignment of *SiDIV1A* and *SiDIV1B* with *AmDIV* (snapdragon). Two MYB domains are indicated. (B) The tree with *AmDIV* as outgroup indicates that Gesneriaceae *DIV* homologs appear to duplicate into *DIV1* and *DIV2* clades but in African violet the *DIV1* further duplicated into *SiDIV1A* and *SiDIV1B*.

**FIGURE S12| qRT-PCR expression of *SiRAD1, SiRAD2, SiDIV1A, and* *SiDIV1B* in dissected petals of African violet.** The expressions levels were compared between dorsal, lateral, and ventral petals at stage 9 (black bar), 12 (gray bar), and 15 (white bar) of DA, WT, and VA. Each bar represents three biological repeats (mean ± SD) except that ventral petal of DA at stage 9 represent only two biological repeats. The fold change of expression was calculated relative to tissues which has the highest expression level = 1 of each gene separately. We used African violet 18S rRNA as housekeeping control to provide internal calibration of equal amount of RNA across different tissues.

**FIGURE S13| Biological replicates of flower qRT-PCR in WT, DA, and VA**

The expressions levels were compared between dorsal, lateral, and ventral petals at stage 9 (black bar), 12 (gray bar), and 15 (white bar) of DA, WT, and VA. Each bar represents one biological replicates and standard deviation was calculated from three technical repeats of that replicate. All samples have three biological repeats (three bars) excepting that ventral petal of DA at stage 9 have only two biological repeats. The fold change of expression was calculated relative to the transcript amount of *SiCYC1A* in stage 9 dorsal petals of WT (the first biological replicate). We used African violet 18S rRNA as housekeeping control to provide internal calibration of equal amount of RNA across different tissues.

# Supplementary Tables

**Supplementary Table 1| Stages of flower development in African violet**

| Stage | Morphology |
| --- | --- |
| 1 | Dome shape: the floral meristem exhibits a hemispherical dome form |
| 2 | Pentagon shape: establishment of the five fold symmetry is completed; the floral primordial is not visible |
| 3 | The sepals initiate as a protrusion at the points of pentagon |
| 4 | The sepals are differentiated; however the floral meristem remains flat |
| 5 | The petals and stamens initiate simultaneously |
| 6 | The petals and stamens differentiated, and the pistil initiates |
| 7 | The petals grow and enfold the inner whorls  The trichomes are visible on the abaxial part of petals |
| 8 | The sepals open and the petals can be easily seen in the middle of bud |
| 9 | The petal whorl is as large as sepal whorl. The stamens are white and unmatured |
| 10 | The petals are emerged out of the sepals. The stamens are turning whitish-yellow |
| 11 | The yellow stamens mature |
| 12 | The flower bud is going to open |
| 13 | The flower bud is opening |
| 14 | The flower is opened |
| 15 | The flower petals keep expansion |
| 16 | The flower is fully open and the petals stop growing. The stamens ready to dehiscent |

**Supplementary Table 2| List of sequences and accession numbers of *CYC*, *RAD*, and *DIV* for reconstruction gene phylogeny.**

| Gene | Family | Species | Gene Name | NCBI Accession Number |
| --- | --- | --- | --- | --- |
| *CYC* |  | *Calceolaria arachnoidea* | *CaCYC1* | AY423143 |
|  |  | *Calceolaria arachnoidea* | *CaCYC2* | AY423144 |
|  | Plantaginaceae | *Antirrhinum majus* L. | *AmCYC* | Y16313 |
|  | Gesneriaceae | *Bournea leiophylla* | *BlCYC1* | EF486283 |
|  |  | *Bournea leiophylla* | *BlCYC2* | EF486284 |
|  |  | *Chrita heterotricha* Merr. | *ChCYC1C* | JX020500 |
|  |  | *Chrita heterotricha* Merr. | *ChCYC1D* | JX020501 |
|  |  | *Chrita heterotricha* Merr. | *ChCYC2A* | JX020502 |
|  |  | *Chrita heterotricha* Merr. | *ChCYC2B* | JX020503 |
|  |  | *Opithandra dinghushanensis* | *OdCYC1C* | FJ710518 |
|  |  | *Opithandra dinghushanensis* | *OdCYC1D* | FJ710519 |
|  |  | *Opithandra dinghushanensis* | *OdCYC2A* | FJ710520 |
|  |  | *Opithandra dinghushanensis* | *OdCYC2B* | FJ644637 |
|  |  | *Streptocarpus rexii* | *SrCYC1A* | MG989469 |
|  |  | *Streptocarpus rexii* | *SrCYC1B* | MG989470 |
|  |  | *Saintpaulia velutina* | *SvCYC1A* | EF127811 |
|  |  | *Saintpaulia velutina* | *SvCYC1B* | EF127812 |
|  |  | *Saintpaulia ionantha* | *SiCYC1A WT* | **MH129027** |
|  |  | *Saintpaulia ionantha* | *SiCYC1A DA* | **MH129028** |
|  |  | *Saintpaulia ionantha* | *SiCYC1A VA* | **MH129029** |
|  |  | *Saintpaulia ionantha* | *SiCYC1B WT* | **MH129030** |
|  |  | *Saintpaulia ionantha* | *SiCYC1B DA* | **MH129031** |
| *RAD* |  | *Saintpaulia ionantha* | *SiCYC1B VA* | **MH129032** |
|  | Plantaginaceae | *Antirrhinum majus* L. | *AmRAD* | AY954971 |
|  | Gesneriaceae | *Bournea leiophylla* | *BlRAD* | EF207557 |
|  |  | *Conandron ramondioides* | *CrRAD1* | **MH366527** |
|  |  | *Conandron ramondioides* | *CrRAD2* | **MH366528** |
|  |  | *Saintpaulia velutina* | *SiRAD1 WT* | **MH129033** |
|  |  | *Saintpaulia ionantha* | *SiRAD1 DA* | **MH129034** |
|  |  | *Saintpaulia ionantha* | *SiRAD1 VA* | **MH129035** |
|  |  | *Saintpaulia ionantha* | *SiRAD2 WT* | **MH129036** |
|  |  | *Saintpaulia ionantha* | *SiRAD2 DA* | **MH401651** |
|  |  | *Saintpaulia ionantha* | *SiRAD2 VA* | **MH129037** |
| *DIV* | Plantaginaceae | *Antirrhinum majus* L. | *AmDIV* | AY077453 |
|  |  | *Antirrhinum majus* L. | *AmDVL1* | AY077454 |
|  | Gesneriaceae | *Bournea leiophylla* | *BlDIV1* | EF211118 |
|  |  | *Bournea leiophylla* | *BlDIV2* | EF211120 |
|  |  | *Saintpaulia ionantha* | *SiDIV1A WT* | **MH129038** |
|  |  | *Saintpaulia ionantha* | *SiDIV1A DA* | **MH129039** |
|  |  | *Saintpaulia ionantha* | *SiDIV1A VA* | **MH129040** |
|  |  | *Saintpaulia ionantha* | *SiDIV1B WT* | **MH129041** |
|  |  | *Saintpaulia ionantha* | *SiDIV1B DA* | **MH129042** |
|  |  | *Saintpaulia ionantha* | *SiDIV1B VA* | **MH129043** |
| Actin7 | Gesneriaceae | *Saintpaulia ionantha* | *SiActin7* | **MH401652** |
| Numbers staring with **MH** are isolated from this study. | | | | |

**Supplementary Table 3 | List of sequences and efficiency value of primer pairs for quantitative real-time PCR .**

| Gene | Efficiency value | Primer pairs | Sequences |
| --- | --- | --- | --- |
| *Si18S* | 98.6%^a^ | qSi18S_F | 5’- CCA TAA ACG ATG CCG ACC AG -3’ |
|  |  | qSi18S_R | 5’- AGC CTT GCG ACC ATA CTC C -3’ |
| *SiActin* | 99.5%^b^ | qSiActin_F | 5’- AAT GGT GAA GGC TGG ATT TG-3’ |
|  |  | qSiActin_R | 5’- CGA GGT CGT CCC ACA ATA CT-3’ |
| *SiCYC1A* | 108.2%^a^, 97.7%^b^ | qSiCYC1A_TAT_F1 | 5’- ACT GCG ACT CAA CAA GAC AT -3’ |
|  |  | qSiCYC1A_ALP_R1 | 5’- CAG GAG ACG GTA GGG CGG -3’ |
| *SiCYC1B* | 97.4% ^a^, 95.2%^b^ | qSiCYC1B_HCG_F1 | 5’- ATC ATT GCG GTT TAC CAT CTC CTA -3’ |
|  |  | qSiCYC1B_DQH_R1 | 5’- CCT ATT GAT GAA TTT GTG CTG ATC C -3’ |
| *SiRAD1* | 65.8%^a^ | qSiRAD1_3-41_F5 | 5’- CTG CGG TGA AGC AAG AAA -3’ |
|  |  | qSiRAD1_3-240_R5 | 5’- TGT GAC GAA TAG CAT AAT GGA -3’ |
| *SiRAD2* | 90.2%^a^ | qSiRAD2_3-1_F1 | 5’- GCA AAG GAT GAG AAA TCT GAA G -3’ |
|  |  | qSiRAD2_3-188_R1 | 5’- AAA CAC TGG AAC AAG AAA GC -3’ |
| *SiDIV1A* | 97.6%^a^ | qSiDIV1A_TEE_F2 | 5’- GAC TGA AGA AGA ACA CAA GAG G -3’ |
|  |  | qSiDIV1A_QLS_R2 | 5’- TTC CGC CAG AAA GTT GCC -3’ |
| *SiDIV1B* | 90.2%^a^ | qSiDIV1B_HKF_F1 | 5’- GCA CAA GTT TTT CCT GAT GGG ACT C -3’ |
|  |  | qSiDIV1B_FIR_R1 | 5’- GGA AAG CTG CCT GAT GAA GT -3’ |
| ^a^Efficiency value were used in Figure 3, gene expression in WT, DA, and VA.  ^b^Efficiency value were used in Figure 6, gene expression in F1 hybrids (WT × DA) | | | |

**Supplementary Table 4. List of sequences and accession numbers of *GCYC* duplicates used for detecting selective pressures differences**

| Family | Clade | Species | *GCYC* duplicates | NCBI Accession Number |
| --- | --- | --- | --- | --- |
| Gesneriaceae | African | *Saintpaulia ionantha* | *SiCYC1A* | MG989467 |
|  |  | *Saintpaulia ionantha* | *SiCYC1B* | MG989468 |
|  |  | *Saintpaulia velutina* | *SvCYC1A* | EF127811 |
|  |  | *Saintpaulia velutina* | *SvCYC1B* | EF127812 |
|  |  | *Streptocarpus dunnii* | *SdCYC1A* | AF208339 |
|  |  | *Streptocarpus dunnii* | *SdCYC1B* | AF208335 |
|  |  | *Streptocarpus primulifolius* | *SpCYC1A* | AF208340 |
|  |  | *Streptocarpus primulifolius* | *SpCYC1B* | AF208336 |
|  |  | *Streptocarpus rexii* | *SrCYC1A* | MG989469 |
|  |  | *Streptocarpus rexii* | *SrCYC1B* | MG989470 |
|  | Advanced Asiatic | *Bournea leiophylla* | *BlCYC1* | EF486283 |
|  |  | *Conandron ramondioides* | *CrCYC1C* | MG989475 |
|  |  | *Didymocarpus citrinus* | *DcCYC1C* | AY423158 |
|  |  | *Hemiboea bicornuta* | *HbCYC1C* | MG989478 |
|  |  | *Loxostigma sp.* | *LsCYC1C* | AY423161 |
|  |  | *Lysionotus pauciflorus* | *LpCYC1C* | MG989482 |
|  |  | *Opithandra dinghushanensis* | *OdCYC1C* | FJ710518 |
|  |  | *Oreocharis benthamii* | *ObCYC1* | FJ710517 |
|  |  | *Primulina heterotricha* | *PhCYC1C* | JX020500 |
|  |  | *Primulina tabacum* | *PtCYC1* | AF208328 |

**Supplementary Table 5| The petal area and cell size comparison between African violet cultivars**

| Ratio (V/D) | Petal part | Dorsalized actinomorphy | Wild type | Ventralized actinomorphy |
| --- | --- | --- | --- | --- |
| Petal area |  | 1.07 | 2.16^＃^ | 1.11 |
| Cell size | Distal | 1.17 | 1.17 | 0.87 |
|  | Proximal | 1.00 | 0.89 | 0.90 |
| The cell size (including distal and proximal regions) were calculated by dividing petal area to cell numbers averaged from 5 flowers of each cultivar. The petal area (mm^2^) and cell size (um^2^) of each cultivar were further converted into a ratio between ventral and dorsal petals. ^＃^The high ratio (>2) in Wild type indicates a two times larger the size of ventral petal area than that of dorsal petal area. | | | | |
