# Supplementary figures and images for: Genetic Analysis of Floral Symmetry Transition in African Violet Suggests the Involvement of Trans-acting Factor for CYCLOIDEA Expression Shifts
Source: Front Plant Sci. 2018 Aug 15;9:1008. doi: 10.3389/fpls.2018.01008 (PMC6104639; doi:10.3389/fpls.2018.01008)

(A) Wild type

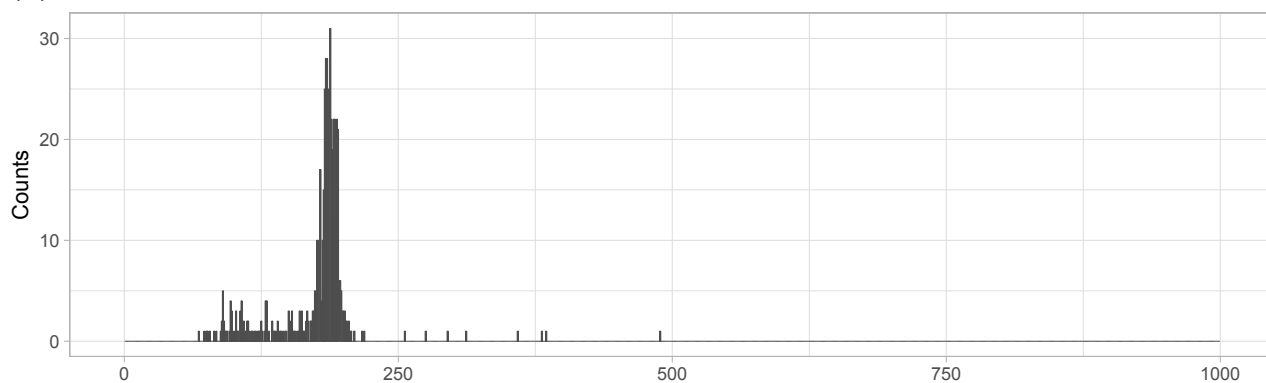

(B) Dorsalized actinomorphy

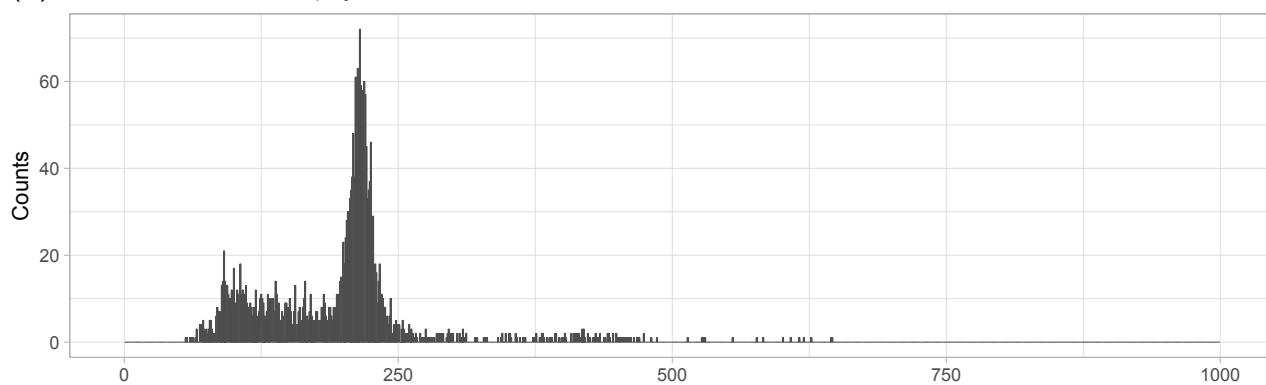

(C) Ventralized actinomorphy

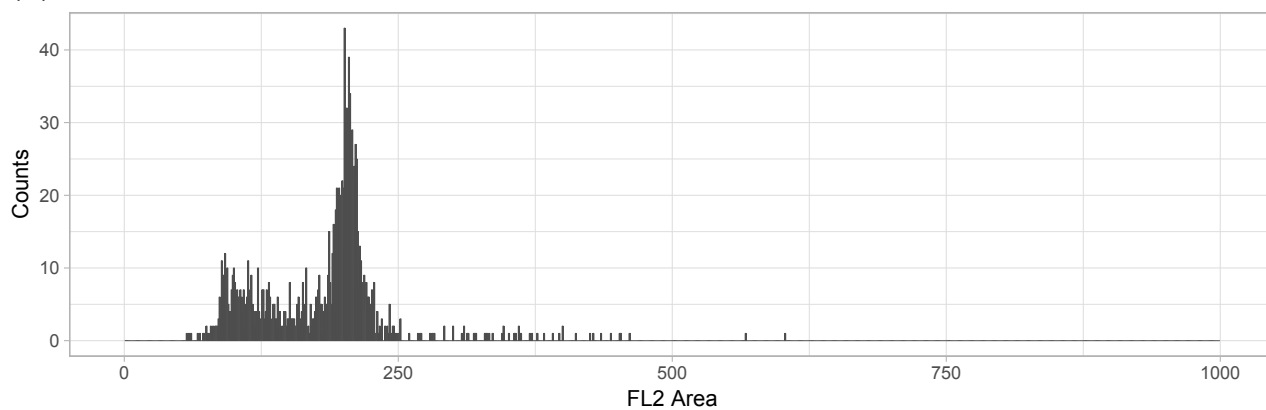

Supplement: Supplementary file 2 [file Image_1.PDF]

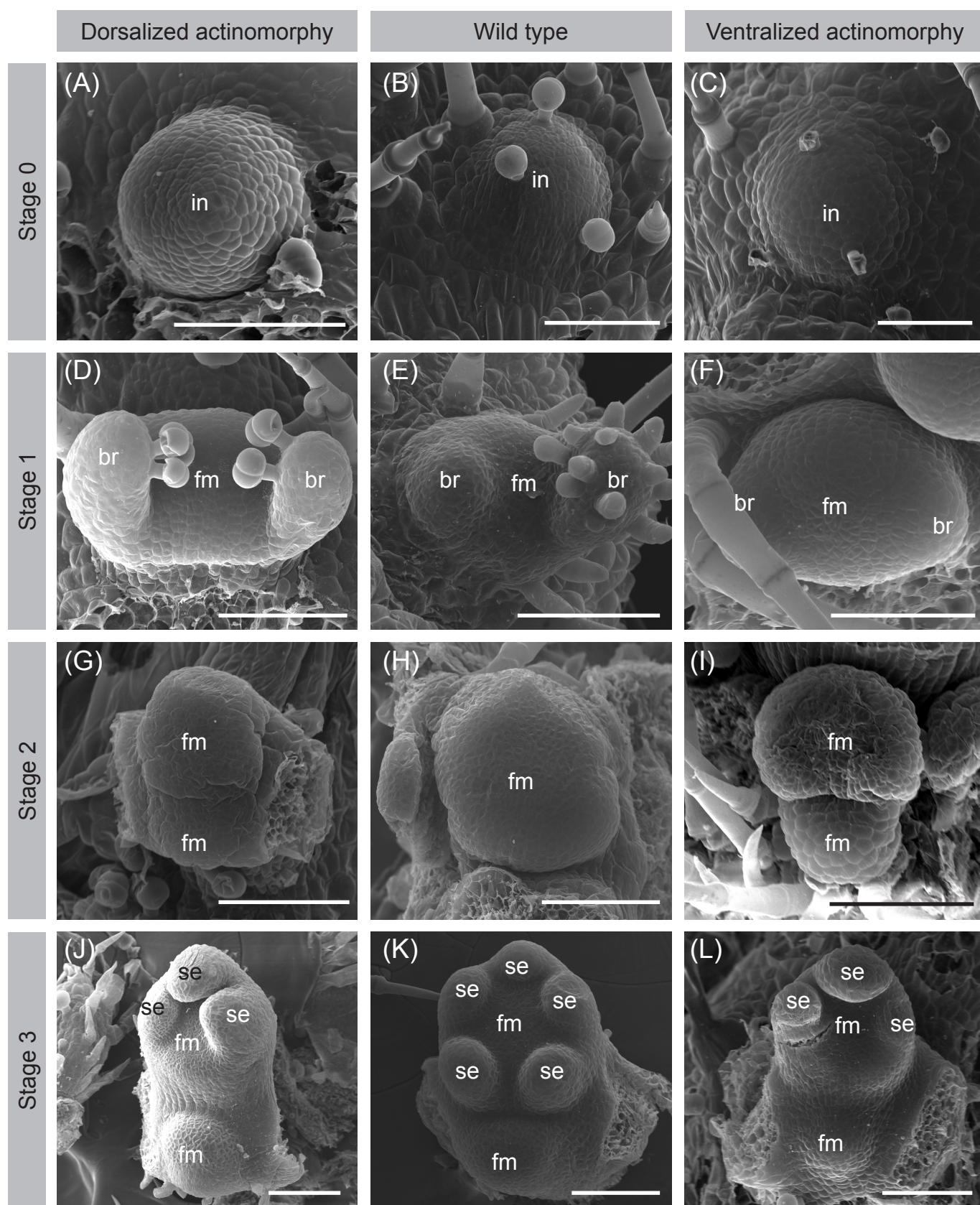

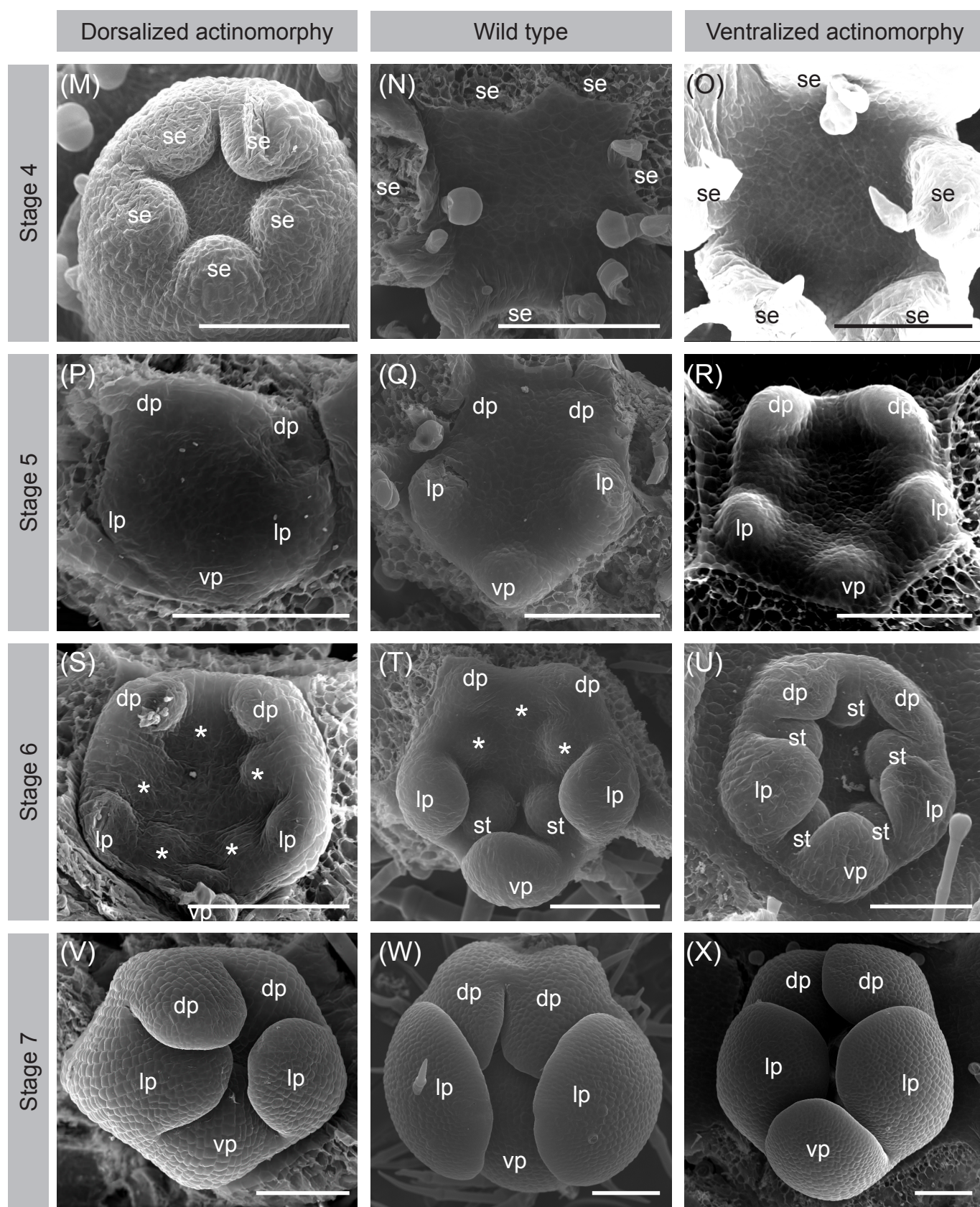

Dorsalized actinomorphy

Wild type

Ventralized actinomorphy

Stage 7 (petal removed)

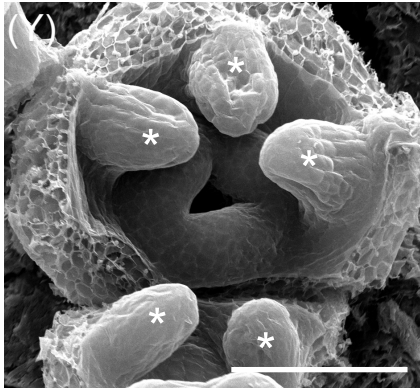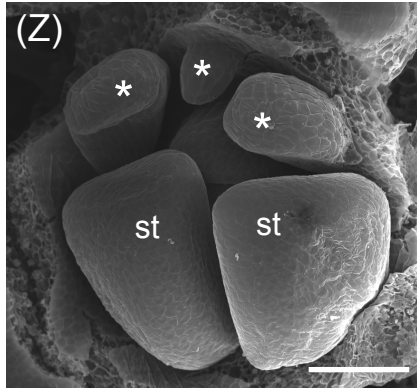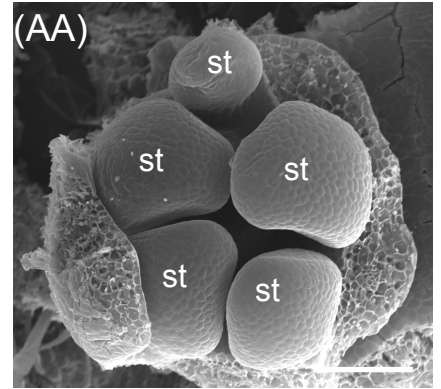

Supplement: Supplementary file 3 [file Image_2.PDF]

(A)

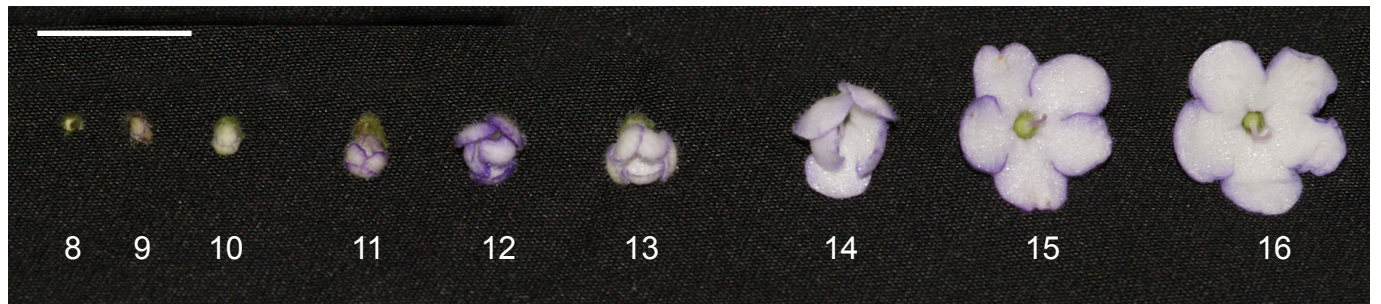

(B)

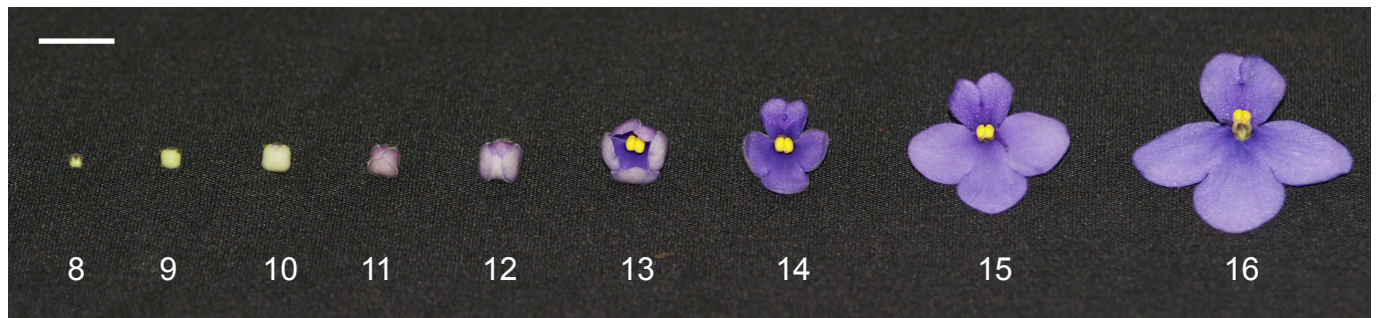

(C)

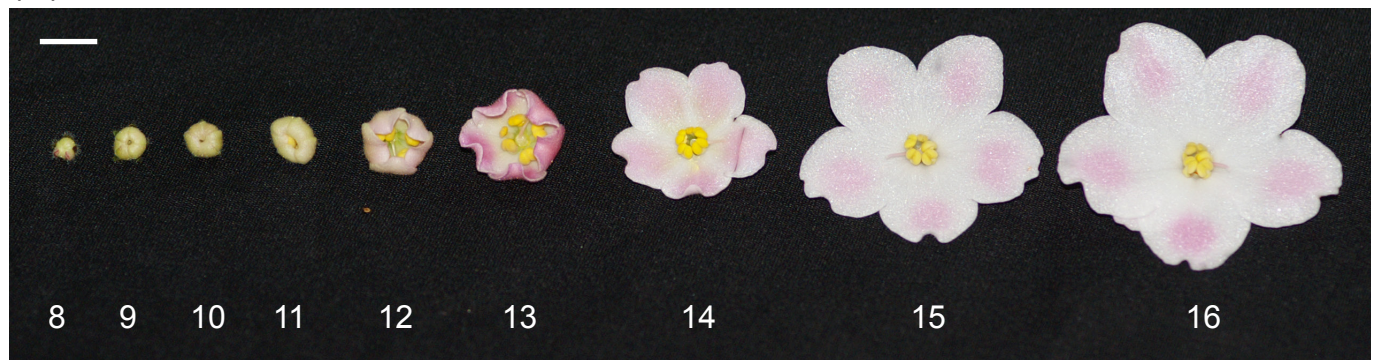

Supplement: Supplementary file 4 [file Image_3.PDF]

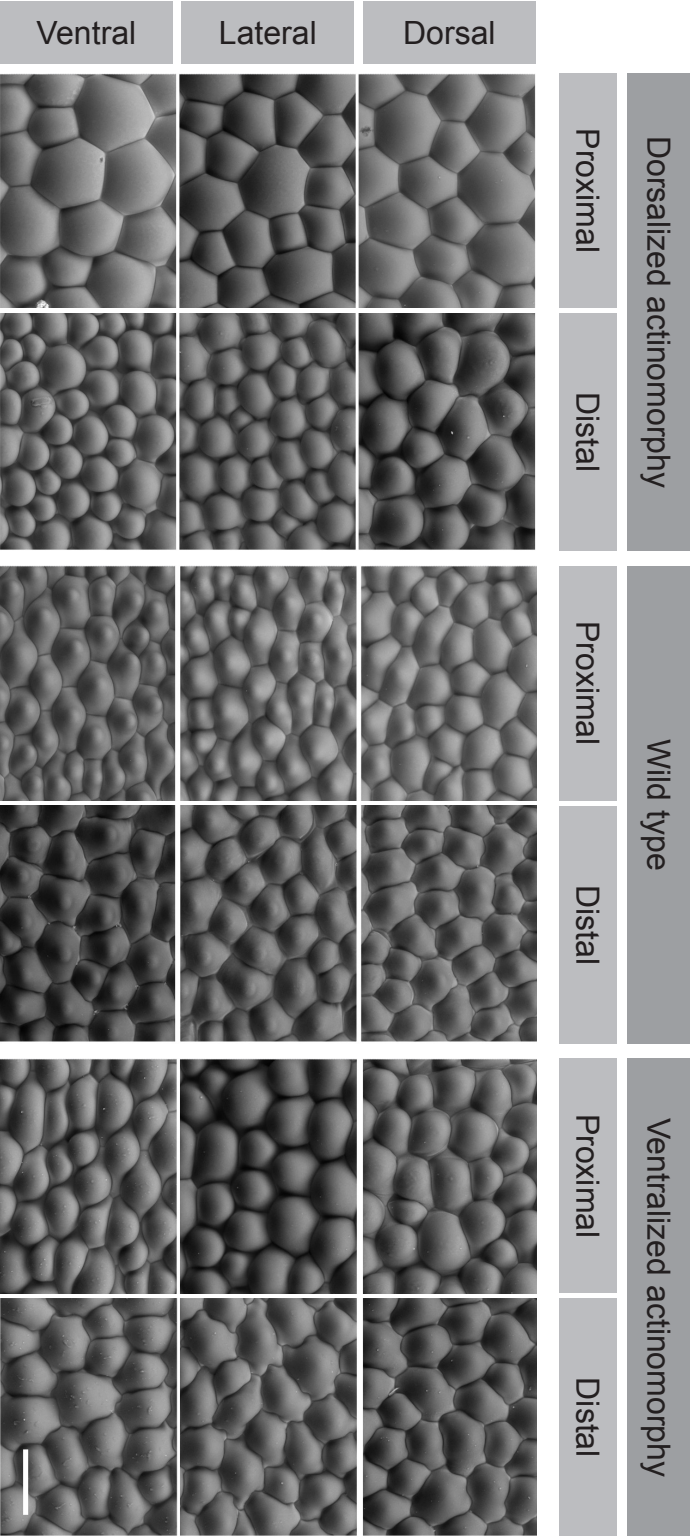

Supplement: Supplementary file 5 [file Image_4.PDF]

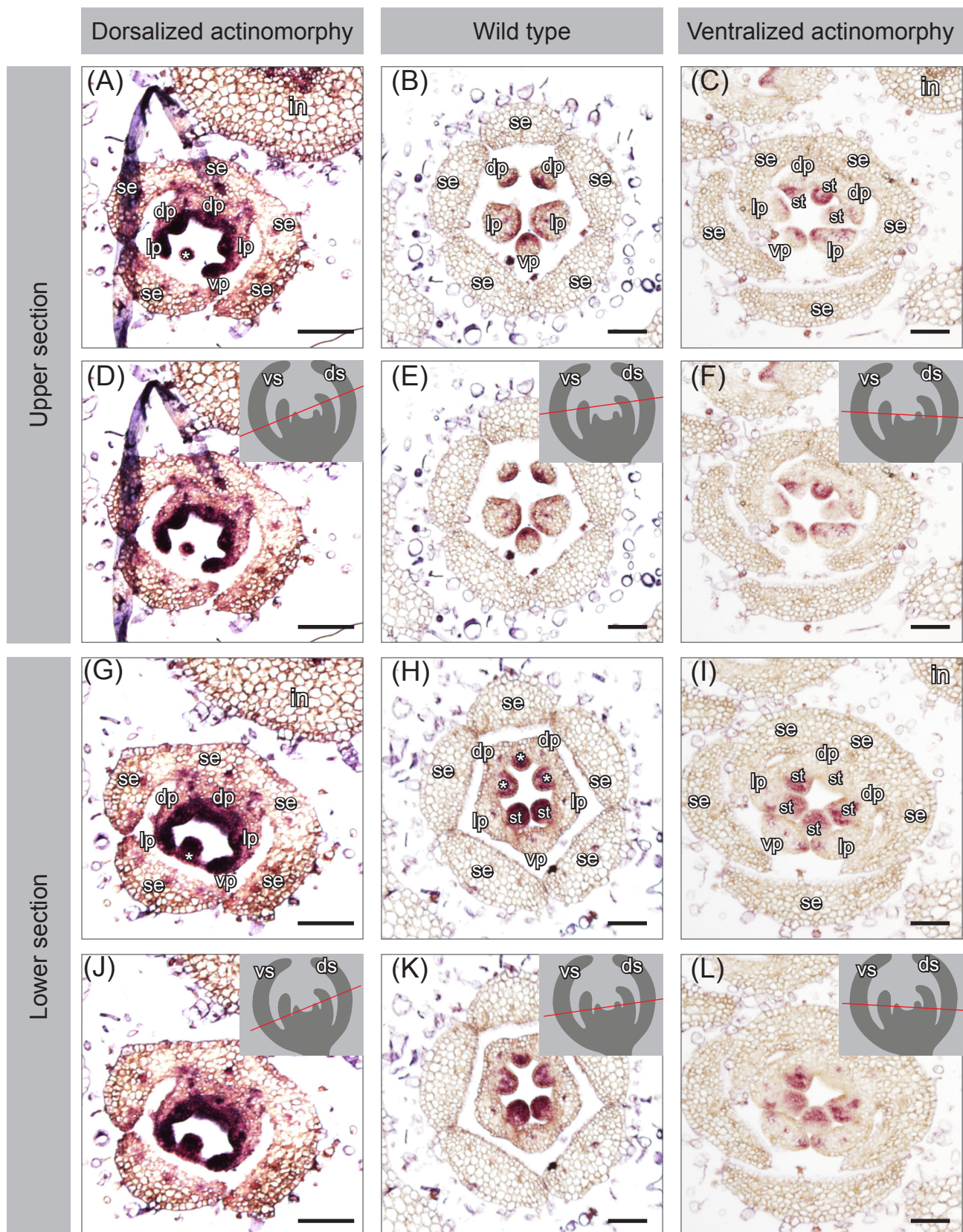

Supplement: Supplementary file 6 [file Image_5.PDF]

(A)

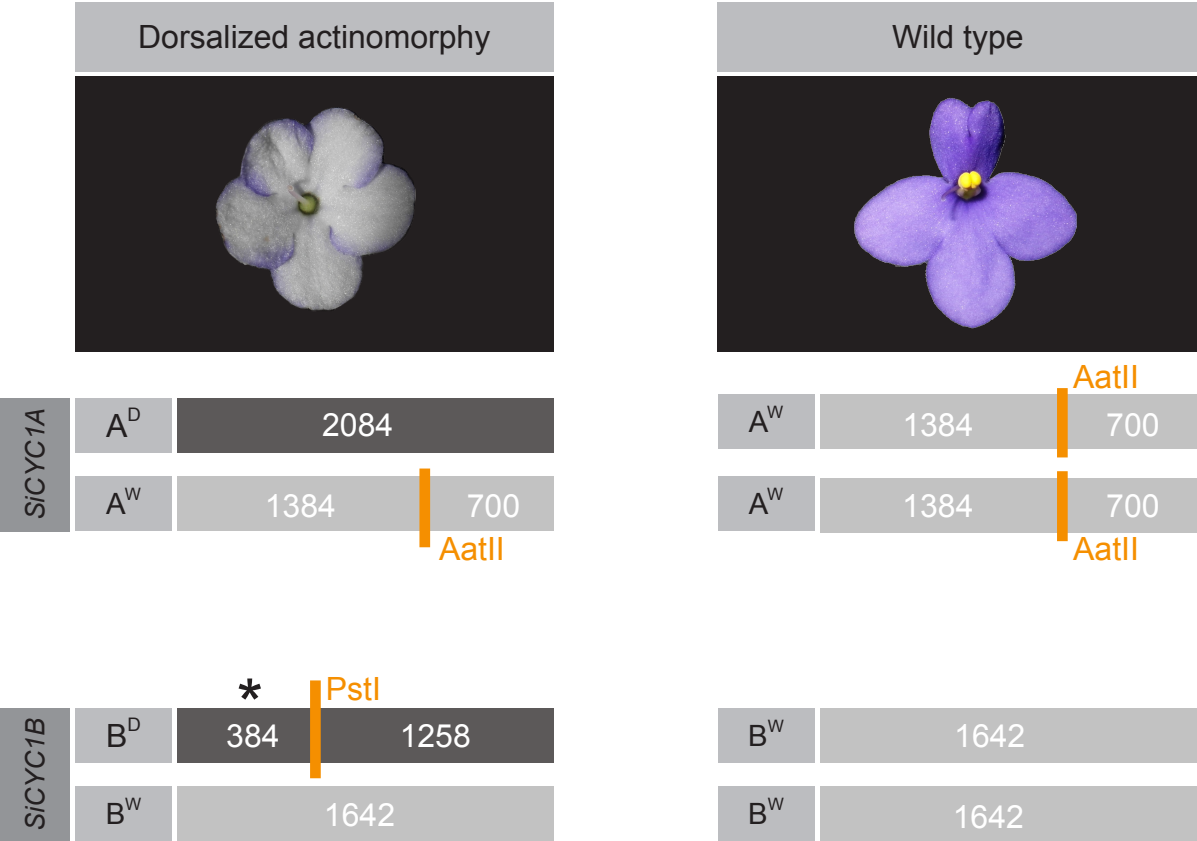

(B)

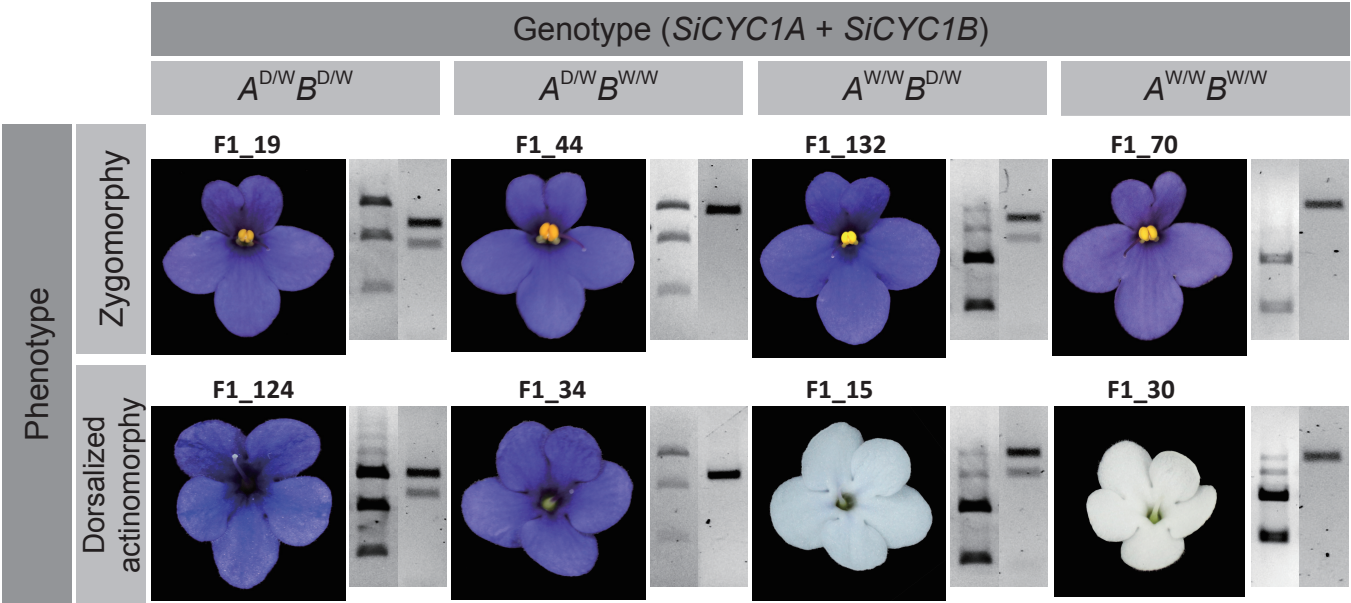

Supplement: Supplementary file 7 [file Image_6.PDF]

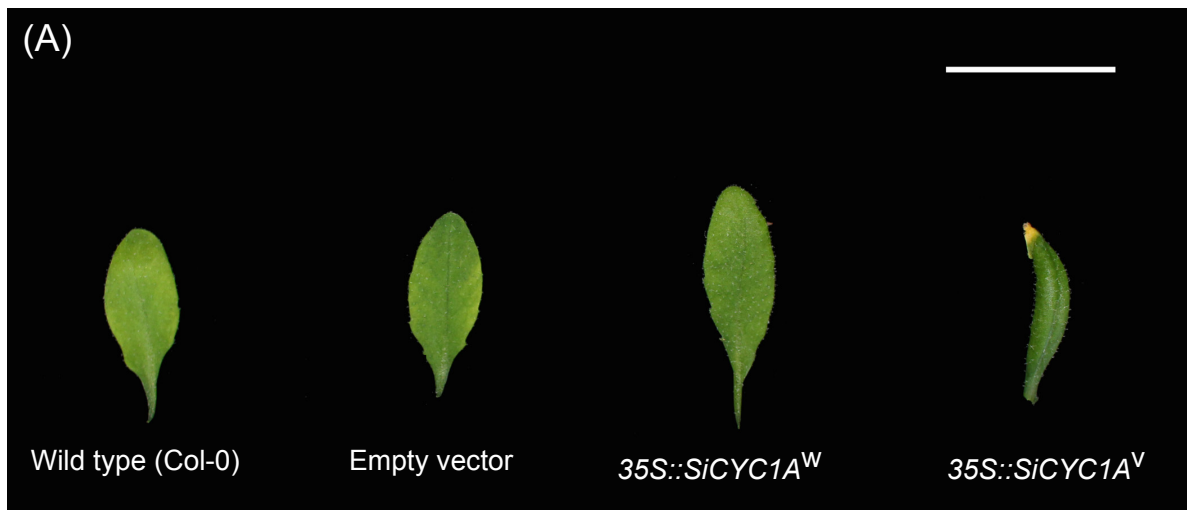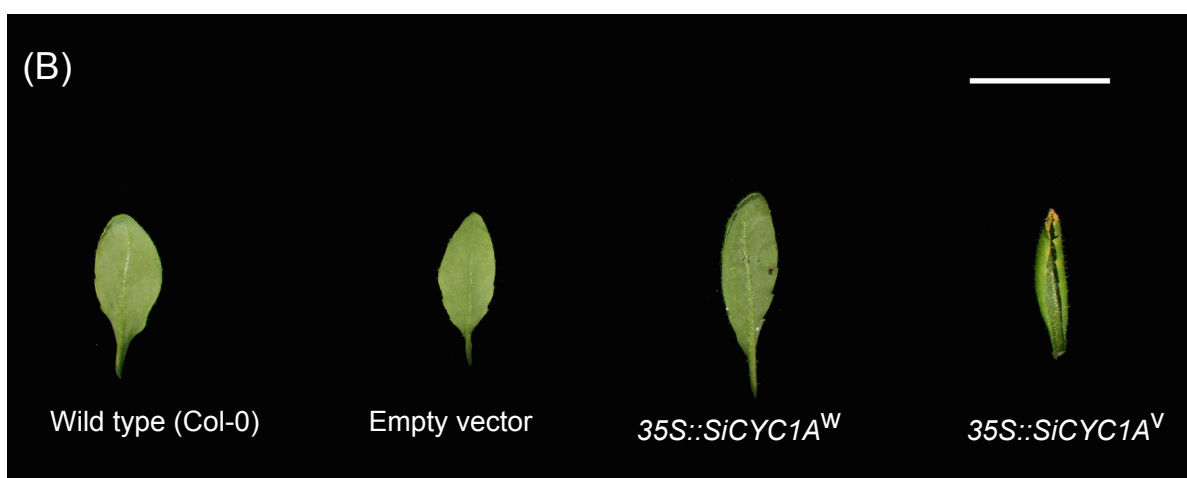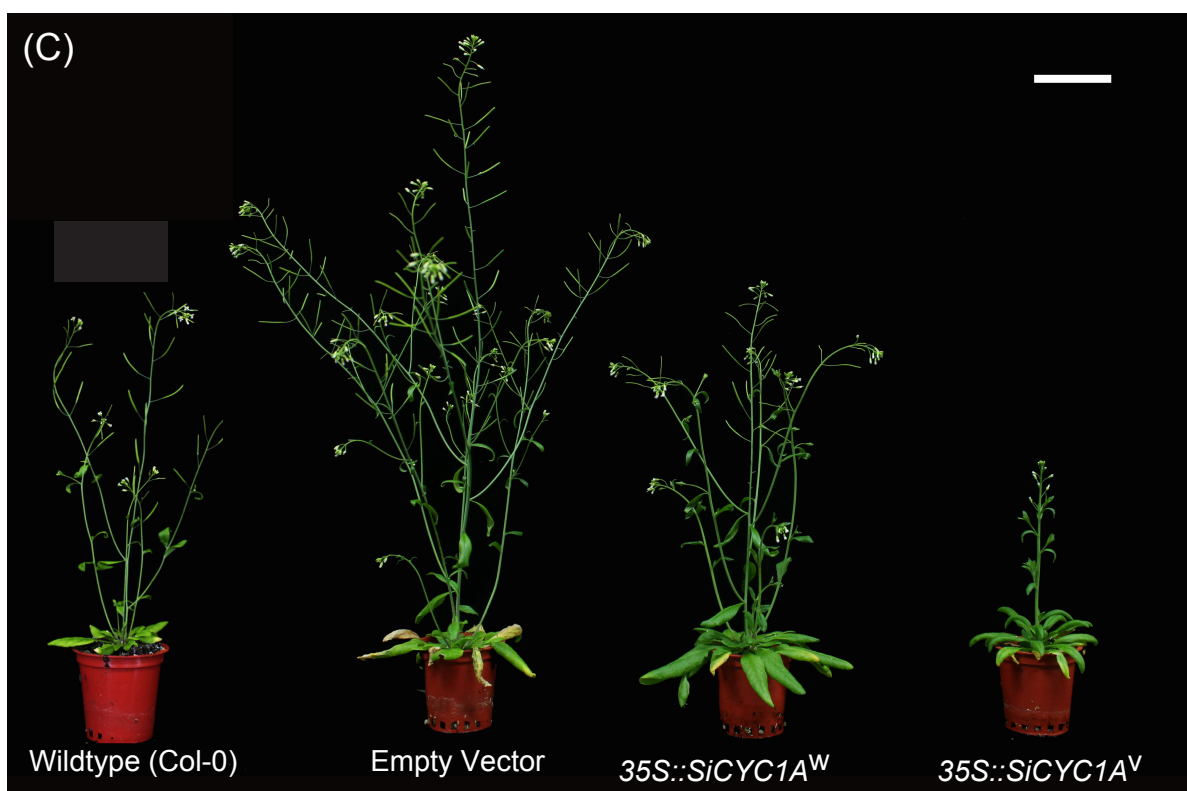

Supplement: Supplementary file 8 [file Image_7.PDF]

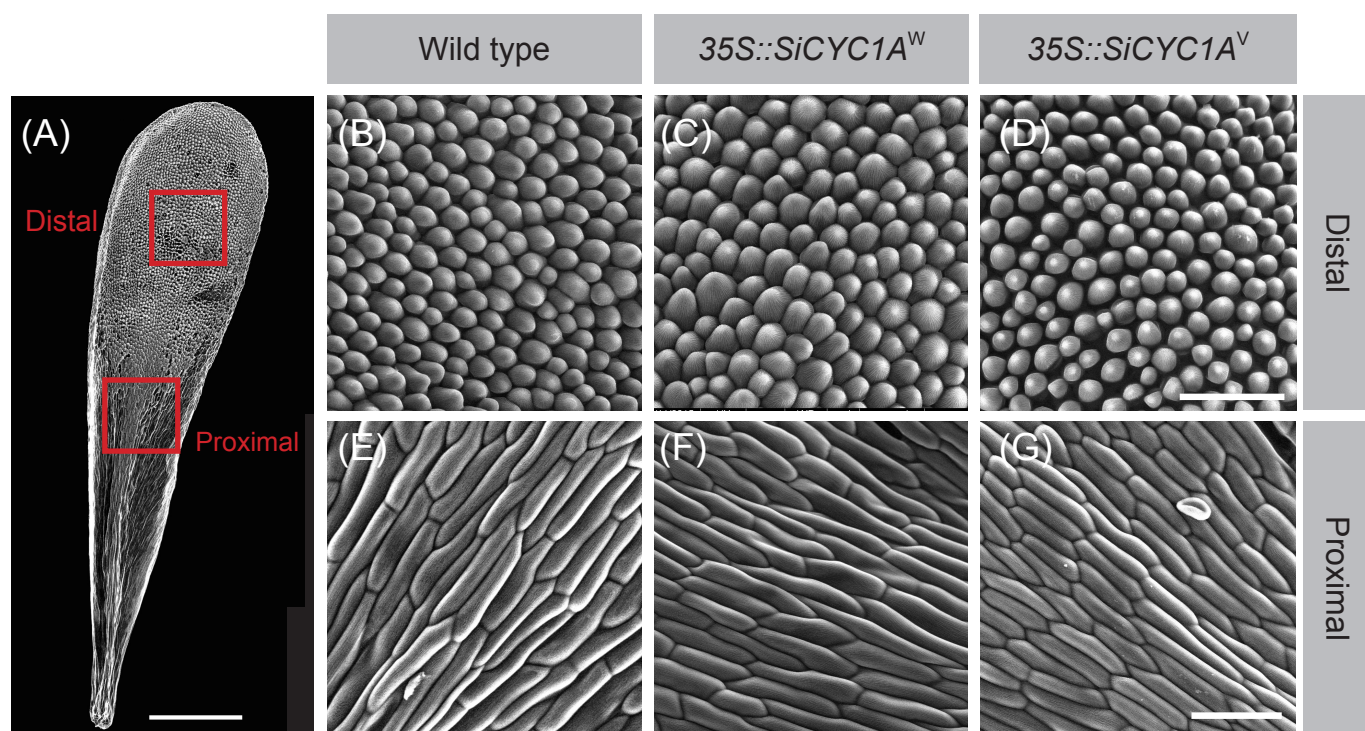

Supplement: Supplementary file 9 [file Image_8.PDF]

F1\_140

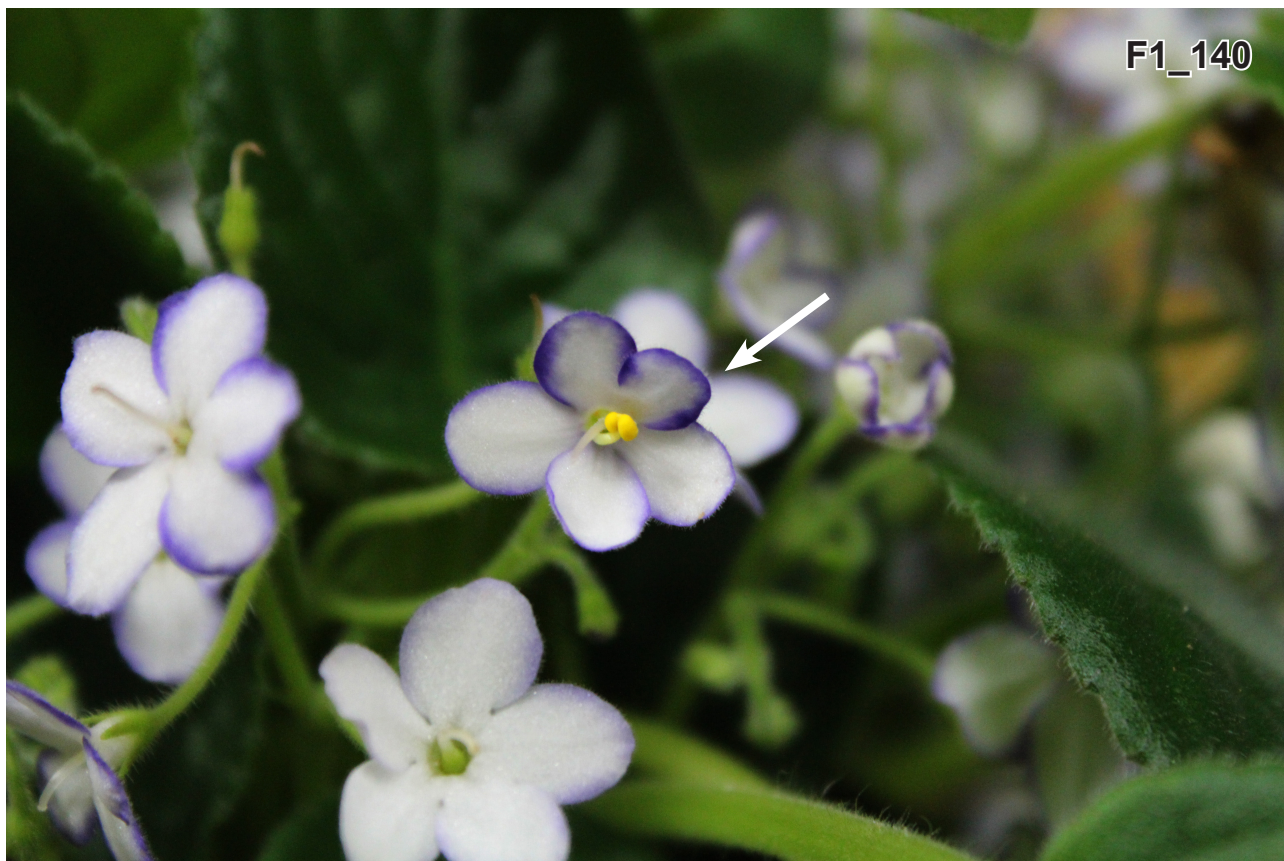

Supplement: Supplementary file 10 [file Image_9.PDF]

(A)

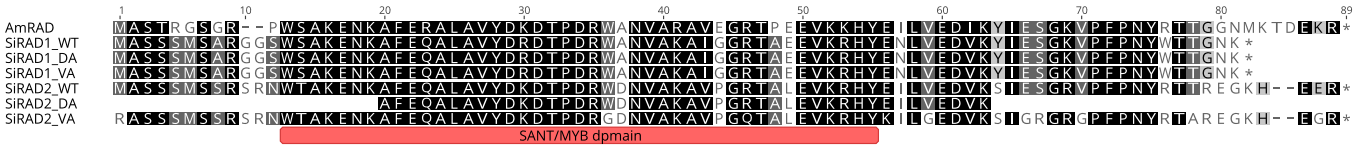

(B)

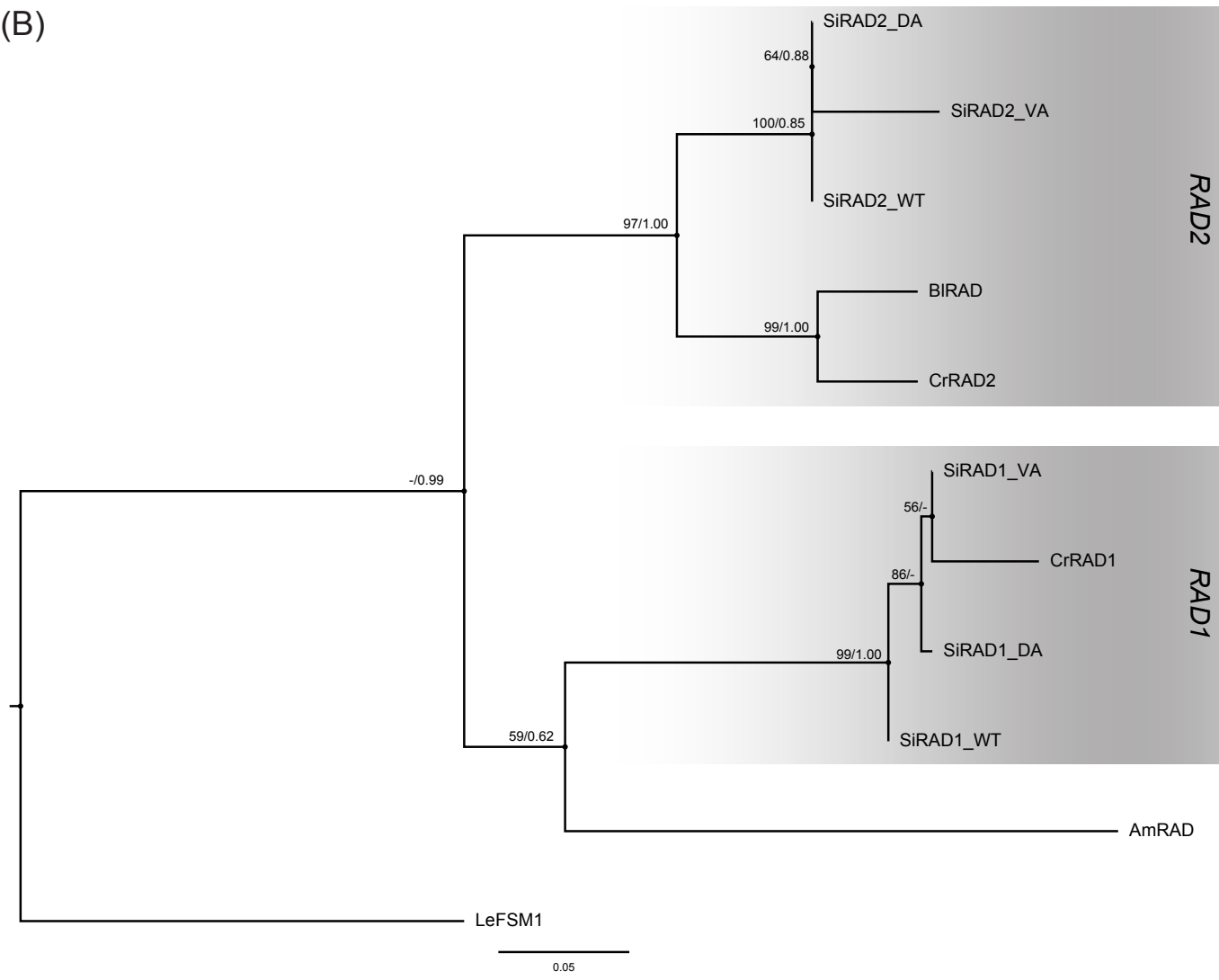

Supplement: Supplementary file 11 [file Image_10.PDF]

(A)

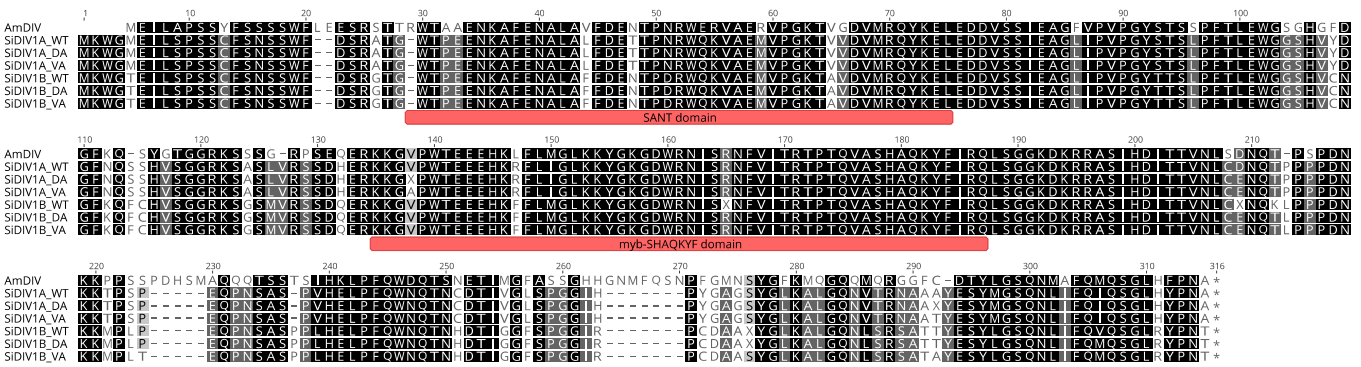

(B)

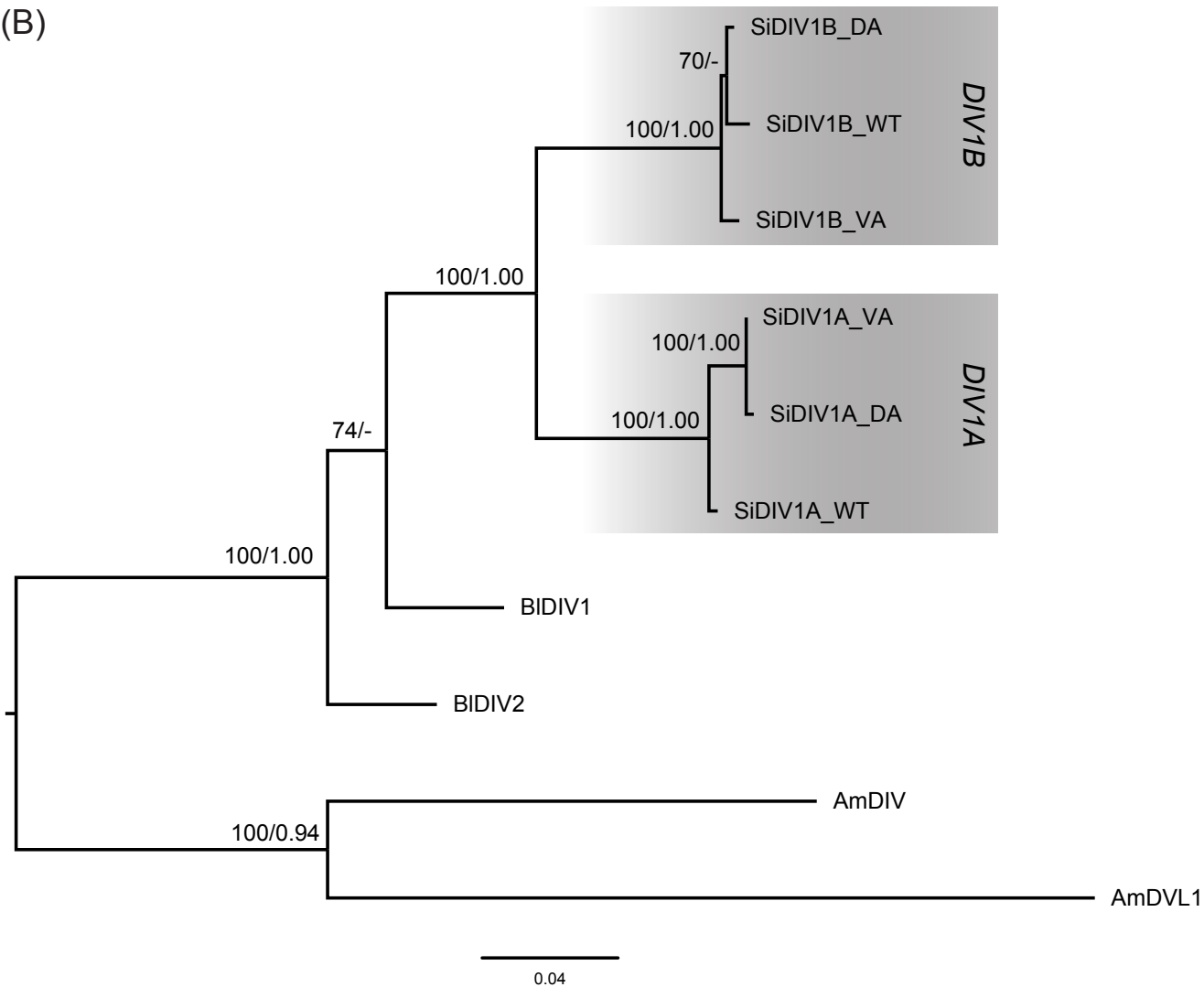

Supplement: Supplementary file 12 [file Image_11.PDF]

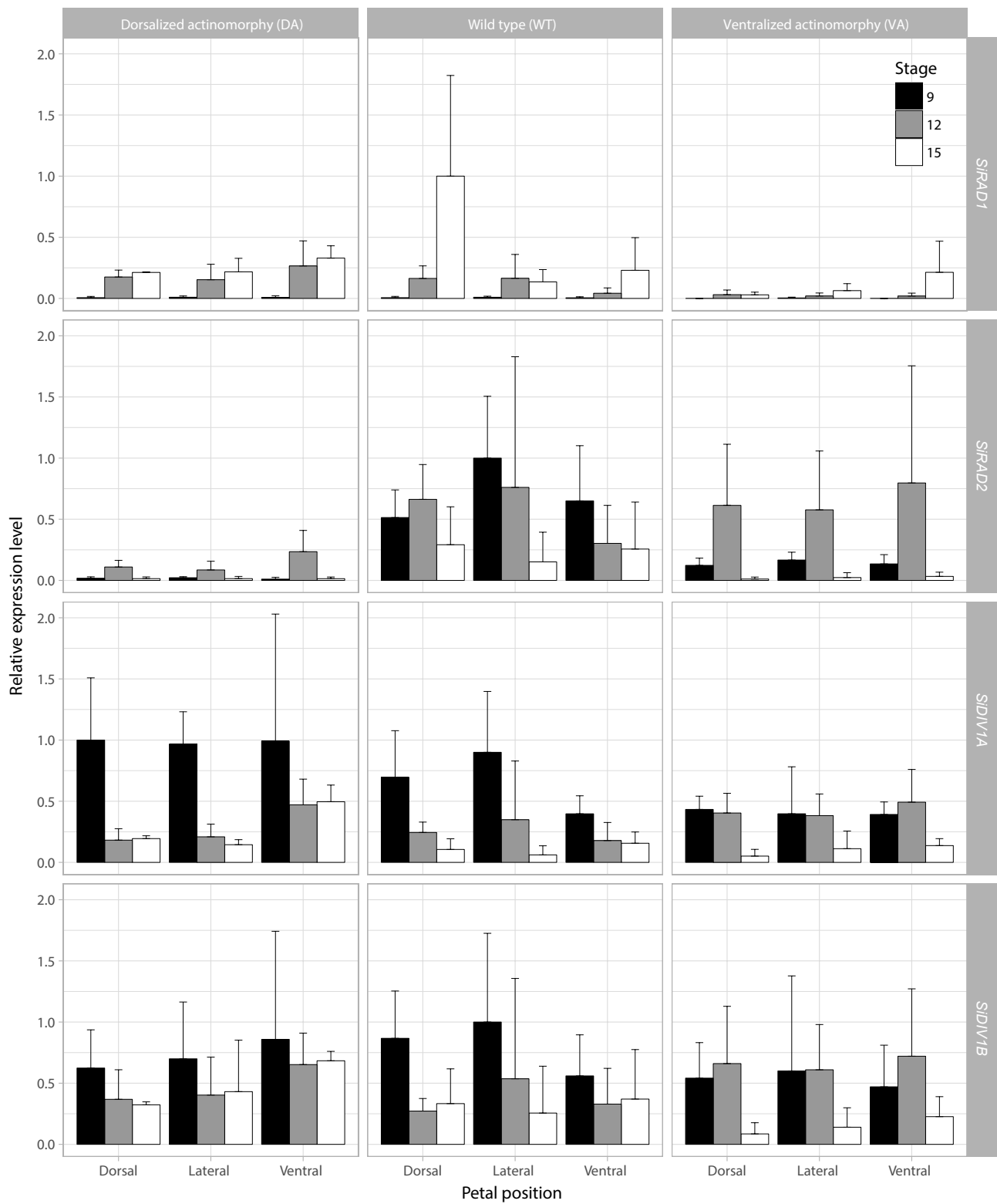

Supplement: Supplementary file 13 [file Image_12.PDF]

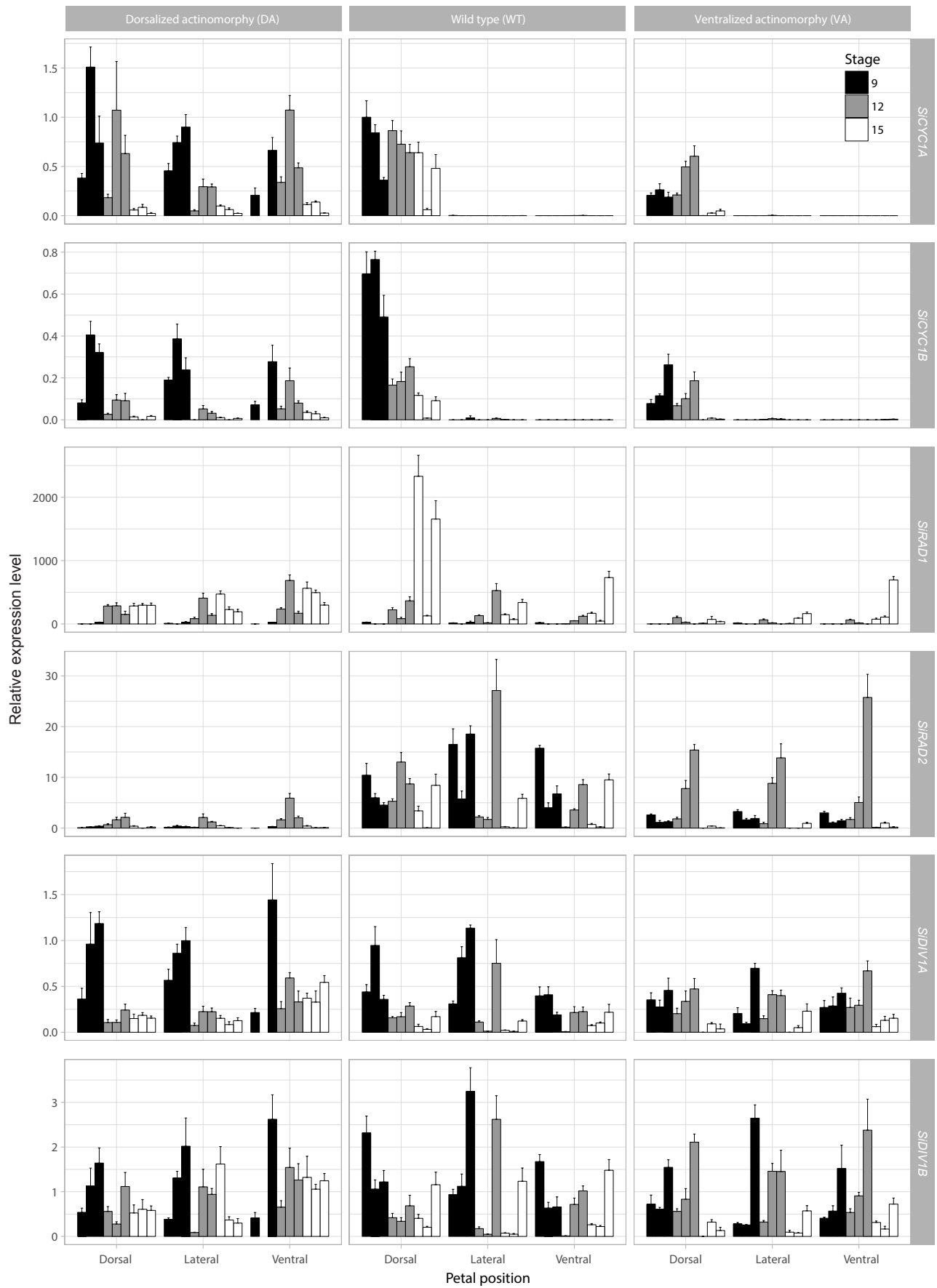

Supplement: Supplementary file 14 [file Image_13.PDF]
